# Knockdown of CENPM activates cGAS-STING pathway to inhibit ovarian cancer by promoting pyroptosis

**DOI:** 10.1186/s12885-024-12296-5

**Published:** 2024-05-01

**Authors:** Wei Xie, Leiying Zhang, Junjing Shen, Fengdi Lai, Wenling Han, Xiaoyan Liu

**Affiliations:** Department of Obstetrics and Gynecology, First Affiliated Hospital of Gannan Medical University, No. 23, Qingnian Road, Zhanggong District, Ganzhou City, Jiangxi Province 341000 China

**Keywords:** Ovarian cancer, CENPM, Hub gene, Pyroptosis, cGAS-STING pathway

## Abstract

**Objective:**

We aimed to screen novel gene signatures for ovarian cancer (OC) and explore the role of biomarkers in OC via regulating pyroptosis using bioinformatics analysis.

**Methods:**

Differentially expressed genes (DEGs) of OC were screened from GSE12470 and GSE16709 datasets. Hub genes were determined from protein–protein interaction networks after bioinformatics analysis. The role of Centromeric protein M (CENPM) in OC was assessed by subcutaneous tumor experiment using hematoxylin–eosin and immunohistochemical staining. Tumor metastasis was evaluated by detecting epithelial-mesenchymal transition-related proteins. The proliferation, migration, and invasion were determined using cell counting kit and transwell assay. Enzyme-linked immunosorbent assay was applied to measure inflammatory factors. The mRNA and protein expression were detected using real-time quantitative PCR and western blot.

**Results:**

We determined 9 hub genes (KIFC1, PCLAF, CDCA5, KNTC1, MCM3, OIP5, CENPM, KIF15, and ASF1B) with high prediction value for OC. In SKOV3 and A2780 cells, the expression levels of hub genes were significantly up-regulated, compared with normal ovarian cells. CENPM was selected as a key gene. Knockdown of CENPM suppressed proliferation, migration, and invasion of OC cells. Subcutaneous tumor experiment revealed that CENPM knockdown significantly suppressed tumor growth and metastasis. Additionally, pyroptosis was promoted in OC cells and xenograft tumors after CENPM knockdown. Furthermore, CENPM knockdown activated cGAS-STING pathway and the pathway inhibitor reversed the inhibitory effect of CENPM knockdown on viability, migration, and invasion of OC cells.

**Conclusion:**

CENPM was a novel biomarker of OC, and knockdown of CENPM inhibited OC progression by promoting pyroptosis and activating cGAS-STING pathway.

**Supplementary Information:**

The online version contains supplementary material available at 10.1186/s12885-024-12296-5.

## Introduction

Ovarian cancer (OC) is a kind of prevalent female cancer, with a very poor prognosis and high fatality rates [1, 2]. It is frequently identified at an advanced stage which has a dismal five-year survival rate [3]. Additionally, OC has a variety of etiological, histopathological, and biological features, characterized by slow-progressing and aggressive tumor growth [4, 5]. In the advanced stages of OC, cancer cells spread from the ovaries to the pelvis, abdomen, lungs, or multiple secondary sites, which severely limits the efficacy of treatment options that cause fatal clinical outcomes [6]. Currently, platinum-based chemotherapy is the mainstream treatment for OC [7]. Although improved surgical techniques and intensive chemotherapy regimens have improved survival in patients with advanced OC over the past two decades, treatment with non-selective cytotoxic drugs often results in severe toxic effects and transient antitumor responses [8]. Additionally, neurotoxicity, neuropathy, and cardiomyopathy caused by chemotherapy are common side effects that affect the normal life of OC patients [9]. Thus, novel treatment targets of OC are urgent to explore.

Centromeric protein M (CENPM) is a member of centromere proteins [10]. Defects in centromere function can lead to loss or destruction of genomic information, resulting in serious and harmful developmental defects or diseases [11]. The most distinctive feature of malignant tumor cells is their ability to continuously divide and proliferate without control, which may be the result of genomic instability caused by chromosomal instability [12]. CENPM promotes cell cycle progression and inhibits apoptosis by regulating P53 signaling pathway [13]. CENPM has been reported in various cancers. In stomach adenocarcinoma, high expression of CENPN can be used as a novel biomarker for stomach adenocarcinoma, and silencing CENPN inhibits gastric adenocarcinoma cell proliferation through cell cycle protein E1 [14]. CENPI is up-regulated in gastric cancer, which can predict poor prognosis and promote tumor cell proliferation and migration [15]. Knockdown of CENPE exerts a driving effect on the progression of hepatocellular carcinoma and is related to poor clinical features [16]. In addition, centromere proteins like CENPA, CENPN, and CENPK have been widely reported in OC [17, 18]. However, the role of CENPM in OC has not been thoroughly investigated.

Pyroptosis is a process of gasdermin (GSDM)-mediated programmed cell death caused by microbial infection, accompanied by activation of inflammatory bodies and release of the pro-inflammatory cytokines [19, 20]. Pyroptosis is associated with the progression of various diseases, especially malignant tumors [21]. Tumors and pyroptosis have a complicated interaction, and the consequences of pyroptosis on cancer vary depending on tissue and genetic background [22]. The role of pyroptosis in OC has been determined. Curaxin CBL0137 induces caspase-3/ GSDME-dependent pyroptosis through the reactive oxygen species /BAX pathway and also has anti-tumor effects on OC cells in vivo [23]. Diethylhexyl phthalate induces pyroptosis through the SLC39A5/NF-κB/NLRP3 axis in granulosa cells, which leads to ovarian dysfunction [24]. BI 2536 is a potent anti-OC drug that inhibits proliferation, blocks cell cycle, induces apoptosis and pyroptosis, and leads to the accumulation of CD8 + T cells at the tumor site [25]. However, whether CENPM affects OC progression by regulating pyroptosis of tumor cells needs further study.

The cyclic guanosine monophosphate-adenosine monophosphate synthase (cGAS)/stimulator of interferon genes (STING) pathway is a crucial pathway to activate the innate immune system [26]. The cGAS-STING pathway, a central cytoplasmic double-stranded DNA sensor, is a key pathway by which innate immunity responds to infection, inflammation, and cancer [27, 28]. Currently, the cGAS-STING pathway has received considerable attention due to its holistic and multifaceted role in cancer immune surveillance [29, 30]. The cGAS-STING pathway is involved in activating or inhibiting the anti-tumor immune response, with the intensity and timing of cGAS-STING pathway activation as well as the type and state of the tumor [31–33]. Nevertheless, whether CENMP interferes with the development of OC through the cGAS-STING pathway remains unclear and needs to be further investigated.

In this investigation, we screened differentially expressed genes (DEGs) based on the datasets associated with OC. Based on the protein–protein interaction (PPI) network constructed by DEGs, we screened out the hub genes related to OC. We assessed the accuracy of the hub genes in predicting and diagnosing OC via comprehensive bioinformatics analysis. CENPM was selected as the research target. Subsequently, we demonstrated the functional involvement and potential pathway of CENPM knockdown in OC progression via pyroptosis at the animal and cellular levels. Herein, we will discuss how CENPM affects the occurrence and progression of OC by regulating pyroptosis. These findings aim to establish CENPM as a potential target biomarker of OC, offering novel perspectives on OC development and prospective therapeutic guidelines for the subsequent focused treatment of OC.

## Materials and methods

### Dataset selection

The gene expression profiles analyzed in this research were retrieved from the Gene Expression Omnibus (GEO) database (https://www.ncbi.nih.gov/geo/). We searched “ovarian cancer” in the GEO database. Datasets were selected based on the following criteria: (i) Microarray or high-throughput sequencing profiling of OC samples from humans or model animals, with control samples provided for comparison; (ii) Analytical reports with sufficient sample size; (iii) the samples were grouped and processed; (iv) The original data could be called. Two microarray datasets, GSE12470 (serous OC) and GSE16709 (serous OC), were selected for subsequent study.

### DEGs analysis

GEO2R (www.ncbi.nlm.nih.gov/geo/geo2r) was employed to obtain original data as well as volcano maps and dataset maps. DEGs were screened out according to the screening criteria of |logFC|≥ 1.1 and adj. *p* ≤ 0.05 (adj. p: *p* value adjusted by Benjamini–Hochberg multiple test). The top 15 DEGs in each dataset were selected to plot a gene heat map by R language. DEGs Venn diagrams for GSE12470 and GSE16709 were obtained using Draw Venn Diagram tool (http://bioinformatics.psb.ugent.be/webtools/Venn/). The common DEGs were screened out for further analysis.

### Functional enrichment analysis of DEGs

The Gene ontology (GO) and Kyoto Encyclopedia of Genes and Genomes (KEGG) enrichment analyses of DEGs were conducted using DAVID (https://david.ncifcrf.gov/). The most significantly enriched DEGs were selected to display and plot bar charts as well as bubble plots.

### PPI network construction and hub gene identification

STRING (https://www.string-db.org/) online tool was used to analyze the DEGs to predict interactions between proteins. PPI networks were constructed according to the confidence interaction score (set at 0.4) and visualized using Cytoscape software (www.cytoscape.org/). Key modules were identified from the PPI network using MCODE (Molecular Complex Detection). Cytoscape plugin CytoHubba Version 0.1 was used to calculate the connectivity of each protein node to screen hub genes.

### Analysis of key gene

Pathway enrichment analysis for the hub genes was performed using Metascape (https://metascape.org/gp/index.html#/main/step1). GO enrichment chord plot was plotted to reveal the relation between the proteins and pathways as well as the change in pathway function. A principal component analysis (PCA) map was drawn using the hub gene expression in the original sample data as a variable to evaluate whether hub genes can distinguish OC from normal controls. R language was employed to draw the ridge plot of hub genes. The diagnostic accuracy of hub genes was evaluated using the receiver operating characteristic (ROC) curve and area under the curve (AUC). The expression profiles of key genes in OC tumors and normal tissues were analyzed by GEPIA (http://gepia.cancer-pku.cn/).

### Cell culture and treatment

Human OC cells, SKOV3 (iCell-h195; iCell Bioscience Inc, Shanghai, China), A2780 (iCell-h004; iCell Bioscience Inc), and human normal ovarian epithelial cells, IOSE80 (iCell-h112; iCell Bioscience Inc) were cultured in DMEM medium containing 100 U/mL penicillin, 100 μg/mL streptomycin, and 10% fetal bovine serum, and incubated in a 37 °C, 5% CO_2_ humidified incubator (Thermo Scientific, Wilmington, DE, USA).

SKOV3 and A2780 cells were divided into 5 groups: Control (no treatment), si-NC group (transfected with siRNA NC), si-CENPM-1 (transfected with siRNA1), si-CENPM-2 (transfected with siRNA2), and si-CENPM-3 (transfected with siRNA3). The siRNA sequence information was listed in Table S1. The CENPM knockdown in OC cells was conducted by lentivirus vector (GeneChem Biotechnology Co. Ltd Shanghai, China) transfection. Transfection was performed according to the instructions of the Lipofectamine 3000 liposome transfection kit (Invitrogen, Carlsbad, CA, USA). The knockdown efficiency was determined by real-time quantitative PCR detection (RT-qPCR) after 48 h of transfection. After that, the CENPM-knockdown cells were cultured with 10 µM of RU.521 (cGAS inhibitor; MedChemExpress, New Jersey, USA) to explore the role of the cGAS-STING pathway in OC.

### Cell viability detection

After transfection, the CCK-8 assay was carried out to detect the proliferation in each group at four time points: 1 d, 2 d, 3 d, and 4 d. Each group of cells was seeded in 96-well plates with cell suspension (1 × 10^4^ cells) and cultured at 37 °C. Then cells were cultured with a maintenance medium (2% FBS + 98% DMEM). Adding 200 μg/mL of cell suspension to each well, the 96-well plate was incubated in the incubator for 24 h. After removing the culture medium, the cells were washed twice with DPBS. Subsequently, 90 μL of maintenance medium and 10 μL of CCK-8 solution were added to each well. Subsequently, cells were incubated in CCK-8 reagent for 2 h, and the absorbance at 450 nm was then detected under a microplate reader.

### Transwell assay

In the cell migration assay, cells (2 × 10^4^) were added into the upper chamber of the transwell insert (Corning Inc, New York, USA) and cultured for 24 h. For the cell invasion assay, the transwell insert was coated with Matrigel (Becton, Dickinson and Company, New Jersey, USA), and cells (2 × 10^4^) were added into the upper chamber. In the lower chamber of the transwell insert, 600 μL of medium containing 15% fetal bovine serum was added, and the cells were cultured for 24 h in an incubator. Then Matrigel and cells in the upper chamber were wiped away, washed with PBS, fixed with 4% paraformaldehyde at room temperature for 15 min, washed again with PBS, and stained with 0.1% crystal violet at 37℃ for 30 min. Photographs were taken to count the number of cells in each field.

### Enzyme-linked immunosorbent assay (ELISA)

The concentrations of IL-6, IL-1β, and TNF-α were detected using ELISA kits (Esebio, Shanghai, China), following the instructions of manufacturer.

### Subcutaneous tumor experiment

Female nude mice (BALB/c-nu; 6–8 weeks old; 18-20 g; SiPeiFu, Beijing, China) were acclimated to the experimental scenarios before model construction and then randomly divided into LV-NC group and LV-CENPM group, with 6 mice in each group. Transfected cells were injected subcutaneously into the right back of mice (5 × 10^6^ per mouse). After 7 d, tumor size was measured weekly. The mice were anesthetized by inhaling 1–1.5% isoflurane mixed with oxygen for 4–5 min and subsequently euthanized by cervical dislocation to obtain the xenografts after 4 weeks. The xenografts were immediately weighed after collection.

All the animals were fed freely and housed in a pathogen-free environment. The procedures for care and use of animals were approved by the Ethics Committee of the First Affiliated Hospital of Gannan Medical University and all applicable institutional and governmental rules regulating the ethical use of animals were followed.

### Hematoxylin–eosin (HE) and immunohistochemistry staining

The xenograft specimen of mice was fixed in 4% paraformaldehyde. After dehydration and hyalinization, the xenografts were embedded in paraffin to prepare the sections for subsequent experiments. The sections were stained with hematoxylin–eosin reagent (Solarbio, Beijing, China), following the instructions. The stained tissue sections were observed under an optical microscope. For immunohistochemistry staining, the paraffin-embedded sections were dewaxed and rehydrated, and antigenic repair was performed with sodium citrate. Sections were incubated with 3% H_2_O_2_ at room temperature to quench endogenous peroxidase activity and closed with BSA for 20 min. After adding primary antibody (Anti-Ki67, 1:400, Abcam) samples were incubated overnight at 4 °C and then incubated for 1 h at 37 °C with a secondary antibody (goat anti-rabbit IgG, 1:200, Abcam). The immunohistochemical images were obtained with an optical microscope.

### RT-qPCR

Total RNA was extracted using the TRIZOL reagent (Invitrogen). After diluting with RNase-free ultrapure water, the concentration of RNA and the absorbance values at 260 nm and 280 nm were measured using a UV spectrophotometer. Reverse transcription was performed to synthesize cDNA templates in a PCR thermocycler, and BI7500 quantitative PCR instrument (Applied Biosystems, Foster City, CA, USA) was applied to RT-qPCR with the procedures as follows: 95 °C for 10 min for initial denaturation, 95 °C for 10 s for denaturation, 60 °C for 30 s for annealing, and 36 cycles in total. β-actin was selected as an internal reference. The obtained Ct values were analyzed using the 2^−ΔΔCt^ method. Each experiment was repeated three times. The primer sequences could be found in Table S1 (Takara Bio Inc, Otsu, Shiga, Japan).

### Western blot

The proteins were extracted and separated by 10% SDS-PAGE. After being transferred onto polyvinylidene fluoride membranes, the membrane was transferred for 2 h at 4 °C and blocked with 5% non-fat milk-TBST, shaking on a room temperature shaker for 2 h. After blocking and washing the polyvinylidene fluoride membrane with washing solution, then the primary antibody was added. The primary antibodies used in this study were as follow: anti-Caspase-1 (1:1000; Abcam, Cambridge, MA, USA); anti-GSDMD (1:1000; Abcam); anti-NLRP3 (1:1000; Abcam); anti-CENPM (1:2000, Abcam) anti-E-cadherin (1:2000, Abcam); anti-N-cadherin (1:2000, Abcam); anti-vimentin (1:2000, Abcam); anti-cGAS (1:1000; Abcam); anti-p-STING (1:1000; Invitrogen); anti-STING (1:1000; Abcam); anti-β-actin (1:2000; Abcam). The membrane was incubated with shaking at 4 °C overnight. Then adding horseradish peroxidase-labeled goat anti-rabbit IgG (1:5000; Abcam), the membrane was incubated at room temperature with shaking for 1 h. Washing the membrane with TBST 3 times, we performed chemiluminescent detection, exposure, and development to analyze the data. The internal reference was β-actin.

### Statistical analysis

The data in this study were processed using GraphPad Prism 8.0 statistical software. Quantitative data were presented as mean ± standard deviation. Comparisons between two groups were performed using t-tests, and comparisons among multiple groups were performed using one-way ANOVA (analysis of variance). Pairwise comparisons after ANOVA were performed using Tukey's multiple comparisons test. *P* < 0.05 suggested a statistically significant difference.

## Results

### Identification of DEGs

**Fig. 1:**
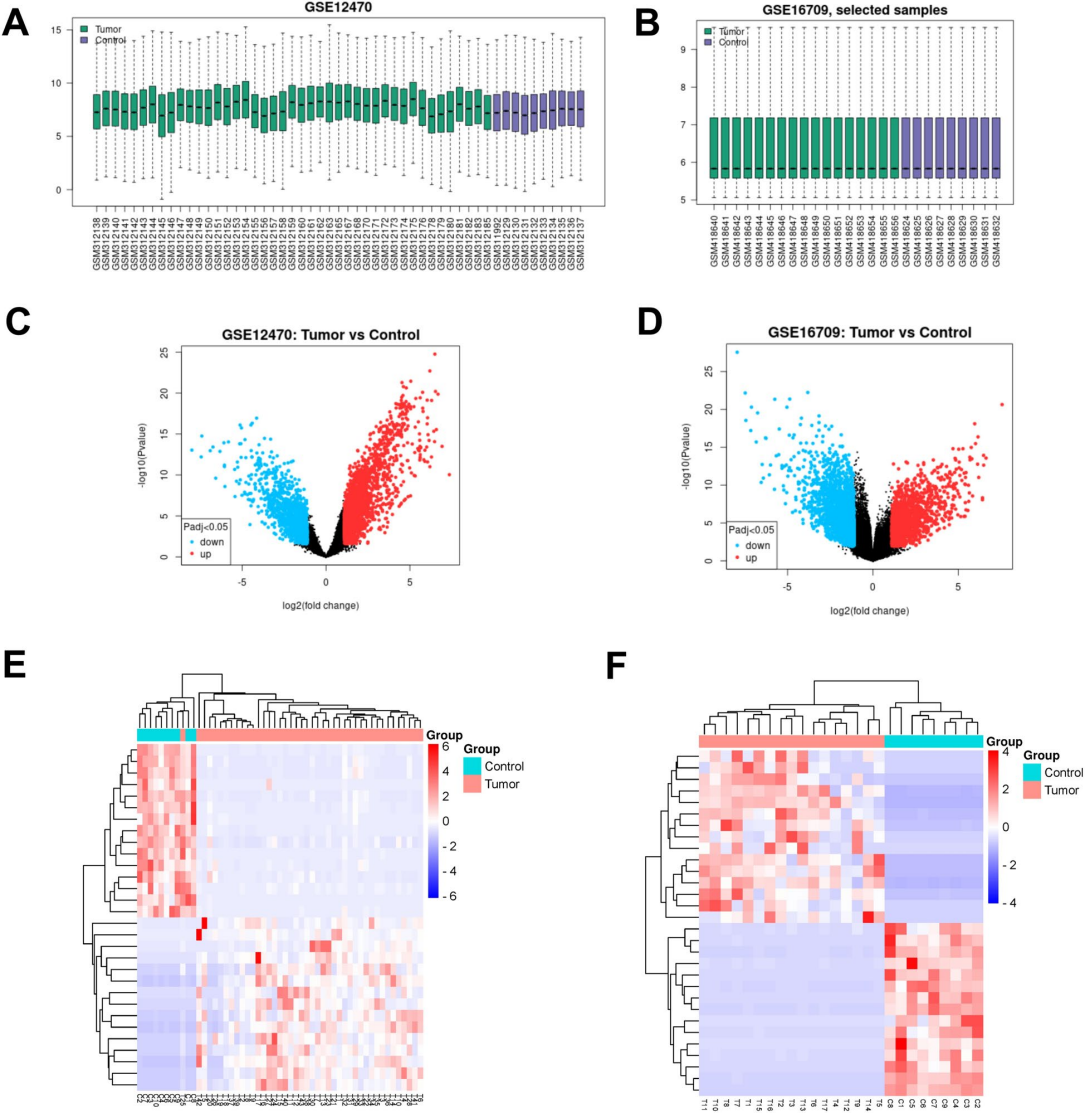
**A**-**B** Cross-comparability evaluation of data set samples; **C**-**D** Volcano diagram: The x-coordinate is log2FoldChange and the y-coordinate is -log10 (*p*-value). Red dots represent up-regulated genes and blue dots represent down-regulated genes; **E** Heat map of GSE12470; F Heat map of GSE16709. Horizontal represents genes, and each column is a sample: red for high-expression genes, blue for low-expression genes

The GSE12470 dataset contained 43 OC tissue samples (8 early-stage tissues and 35 late-stage tissues) and 10 normal samples, while the GSE16709 dataset contained 17 OC tissue samples and 9 normal samples. The gene datasets were standardized and assessed for cross-comparability, suggesting that the selected samples were centered and the numerical distribution met the standard, indicating high microarray data quality and cross-comparability (Fig. 1A, B). Using GEO2R for analysis, we screened 2908 DEGs from the GSE12470 dataset and 3038 DEGs from the GSE16709 dataset, and clustering analysis was performed to obtain volcano plot of DEGs (Fig. 1C-D). The top 15 up-regulated and down-regulated genes were selected (Tables S2-3). The DEGs heatmaps revealed that the samples were clustered well with high confidence (Fig. 1E-F).

Venn diagrams were drawn to screen the common DEGs between the two datasets (Figure S1), demonstrating that there were 869 DEGs in common between the two datasets.

### Function enrichment analysis of common DEGs

**Fig. 2:**
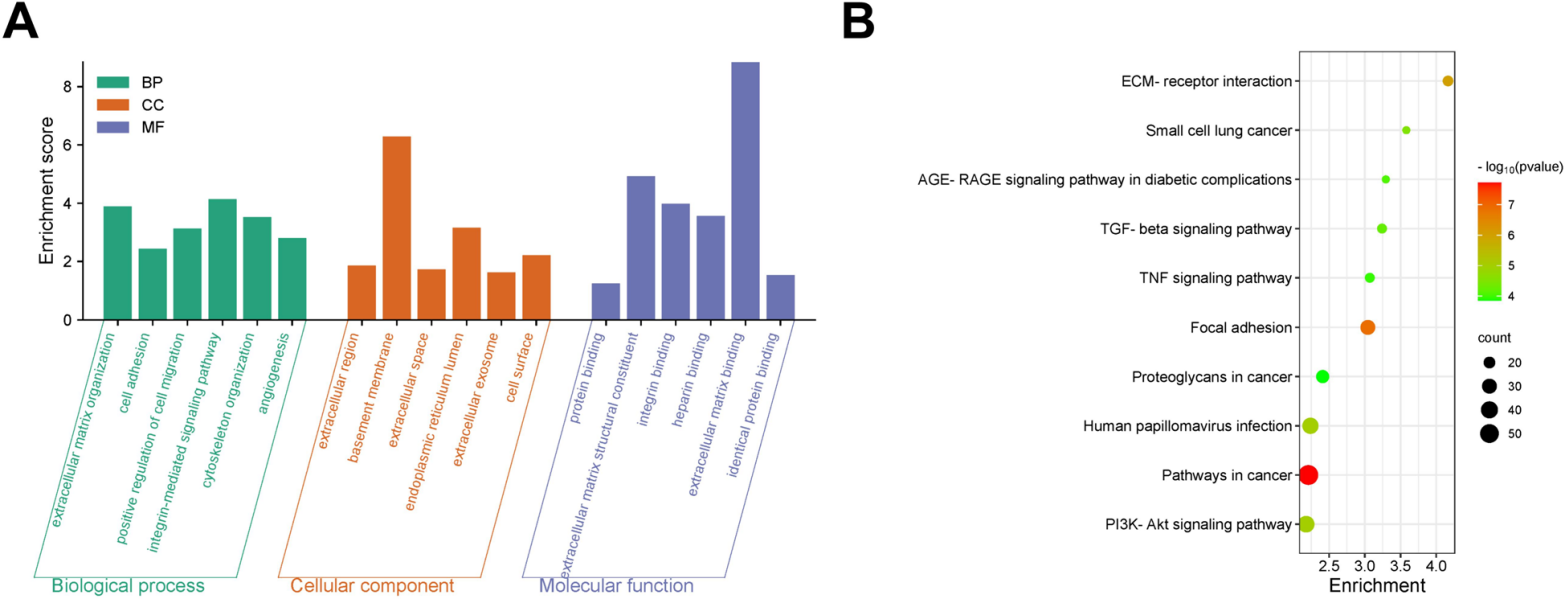
Analysis of common differentially expressed genes (DEGs) **A** Gene Ontology (GO) enrichment analysis the histogram of DEGs, abscissa is GO term, and ordinate is -log10 (*p*-value) of enrichment in each term. **B** Bubble map for Kyoto Encyclopedia of Genes and Genomes enrichment analysis

The results of the GO enrichment analysis of DEGs were divided into 3 parts: molecular function (MF), biological process (BP), and cellular component (CC). The most significantly enriched top 6 GO terms with the minimum *p*-values of each category were displayed in the histogram and the bubble diagram of GO enrichment analysis (Fig. 2A, Figure S2A). The DEGs were significantly enriched in “extracellular matrix organization”, "cell adhesion”, etc. of BP term; “extracellular matrix organization”, “cell adhesion”, etc. of CC term; and “extracellular matrix organization”, “cell adhesion”, etc. of MF term.

According to the KEGG enrichment analysis results of DEGs, the top 10 pathways with the minimum *p*-value, which means the most significant enriched pathway, were selected for display. The results were shown in the histogram and the bubble diagram of KEGG enrichment analysis (Fig. 2B, Figure S2B). The results revealed that the DEGs were mainly enriched in “Pathways in cancer”, “Focal adhesion”, “ECM-receptor interaction”, “PI3K-Akt signaling pathway”, and “Human papillomavirus infection”, etc.

### PPI network construction and hub gene identification

The STRING tool was employed to construct PPI networks based on the DEGs, which were visualized using Cytoscape software (Figure S3). Key modules were identified from the PPI network of DEGs, and Module 1 with the maximum score was selected (Figure S4). Then, genes in Module 1 were sorted according to the score, and the top 9 genes were selected as hub genes for analysis, including KIFC1, PCLAF, CDCA5, KNTC1, MCM3, OIP5, CENPM, KIF15, and ASF1B.

### Analysis of hub genes

**Fig. 3:**
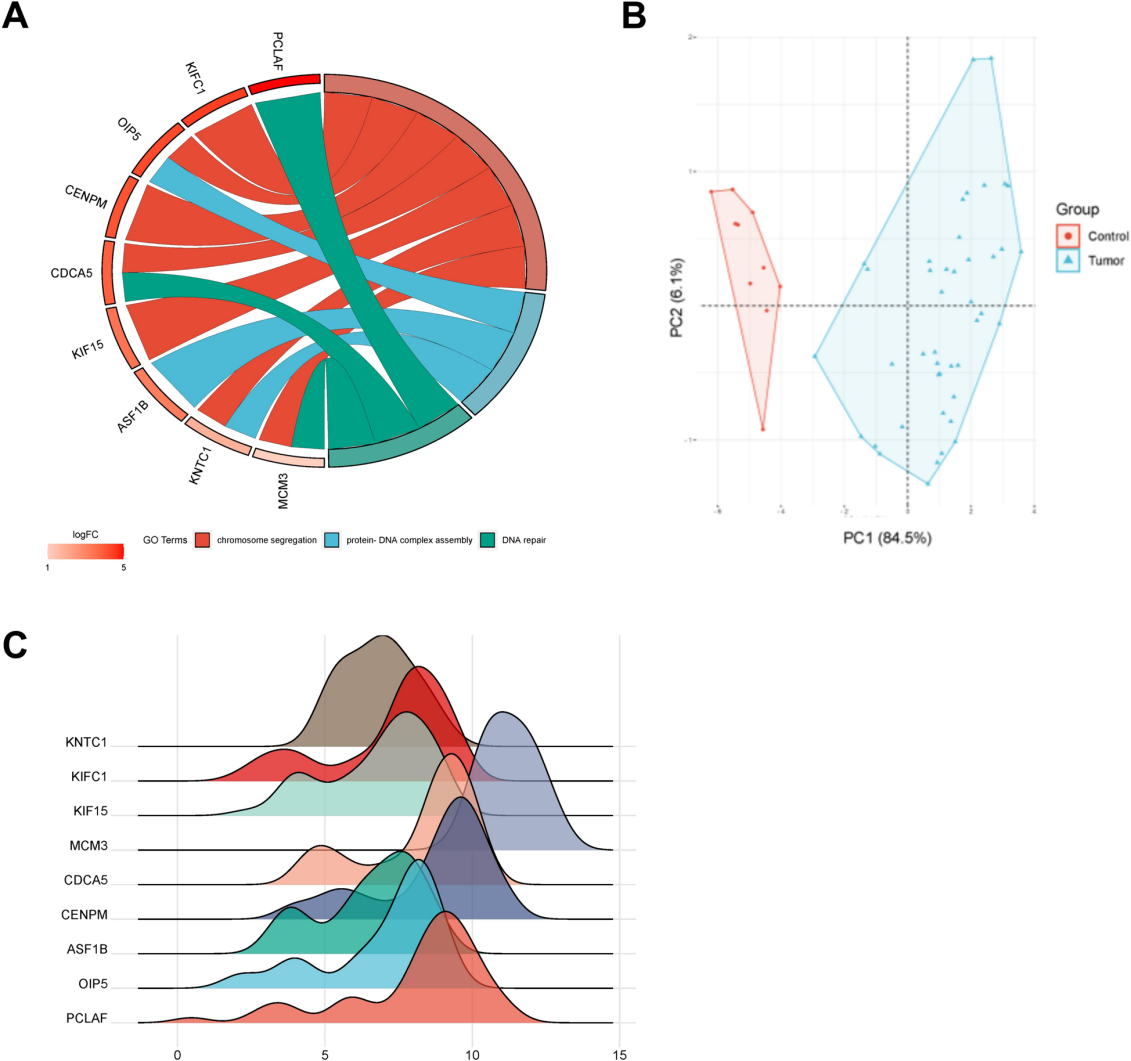
**A** GO enrichment chord map, the data included 3 parts: genes; logFC is the multiple of gene change, used for sequencing and gene block color; The remaining columns are GO term, and different links of the gene indicate whether the gene is in this GO term; **B** Principal component analysis diagram of hub genes, in which axes PC1 and PC2 are the first and second principal components (i.e., explanation rate of difference by potential variables); Dots represent samples, and different colors represent different groups; **C** Hub gene ridge map, horizontal coordinate is gene expression level, mountain height represents sample abundance of corresponding expression level

GO enrichment chord plot reflected the relationship between proteins and pathways, as well as the changes in the pathway functions (Fig. 3A). After processing with R language, we obtained two principal component variables, PC1 and PC2, which provided a total variance explanation rate of 90.6% and could distinguish between the control group samples and OC samples. The scatter plot suggested that the two groups were separated well, and further confirmed the effectiveness of PC1 and PC2 (Fig. 3B). The ridge line plot showed the expression of hub genes (Fig. 3C).

The ROC curve of hub gene was drawn using the original sample data of GSE12470 and GSE16709 datasets (Figures S5, S6). The results demonstrated that the false positive rate of hub genes (KIFC1, PCLAF, CDCA5, KNTC1, MCM3, OIP5, CENPM, KIF15, and ASF1B) testing in GSE12470 was 0.5%, 0%, 0%, 0%, 0%, 0%, 4. 1%, 0%, and 0%, respectively, while the true positive rate was 99.5%, 100%, 100%, 100%, 100%, 100%, 95.9%, 100%, and 100%, respectively. The false positive rate of hub genes in GSE16709 dataset was 3.9%, 2.6%, 15%, 13.7%, 5.6%, 2.6%, 0%, 11.1%, and 13.1%, respectively, while the true positive rate was 96.1%, 97.4%, 85%, 86.3%, 95.4%, 97.4%, 100%, 88.9%, and 86.9%, respectively, indicating that hub genes were good indicators to distinguish OC from healthy controls. The differential expression of hub gene in tumor and non-tumor tissues analyzed by The Cancer Genome Atlas database revealed that the expression level of CENPM in tumor tissues was significantly higher than that in normal tissues (Figure S7).

**Fig. 4:**
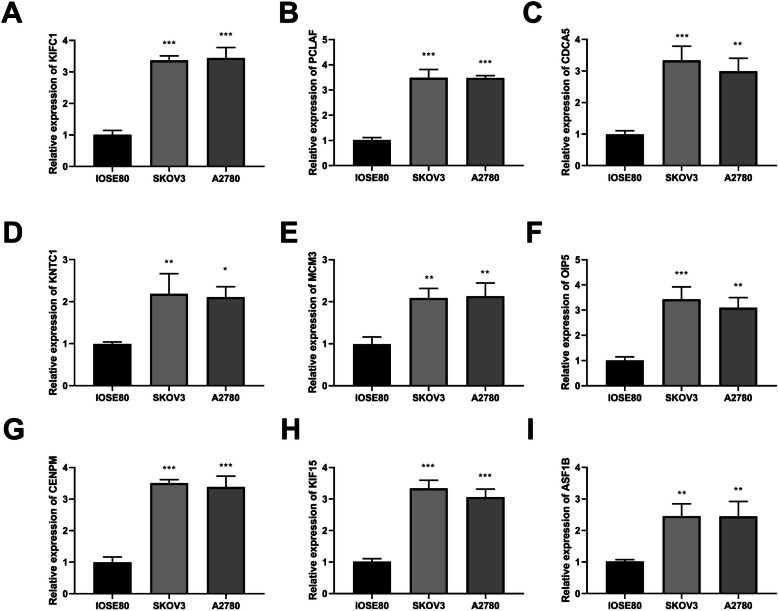
Real-time quantitative PCR (RT-qRCR) detected the mRNA expression levels of KIFC1, PCLAF, CDCA5, KNTC1, MCM3, OIP5, CENPM, KIF15, and ASF1B in cells. ^*^P < 0.05, ^**^P < 0.01, ^***^P < 0.001 *vs.* IOSE80 cells

RT-qPCR results suggested that compared to the normal ovarian cells IOSE80, the mRNA expression levels of KIFC1, PCLAF, CDCA5, KNTC1, MCM3, OIP5, CENPM, KIF15, and ASF1B were significantly up-regulated in SKOV3 and A2780 (Fig. 4).

### Knockdown of CENPM suppresses proliferation, migration, and invasion of OC cells

**Fig. 5:**
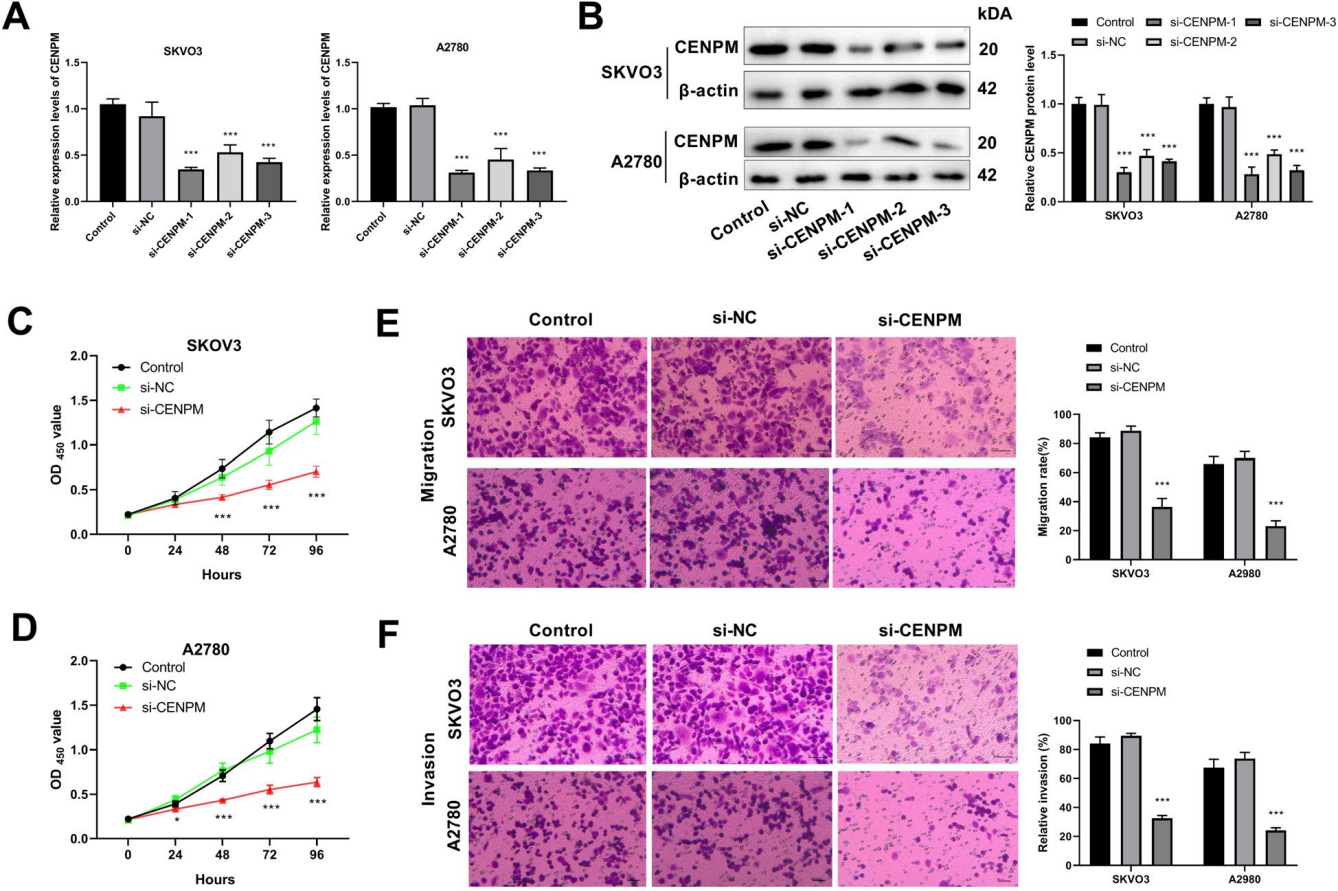
Effect of CENPM on ovarian cancer cell. **A** RT-qPCR was used to detect transfection efficiency; **B** Western blot was used to detect the protein expression level of CENPM; **C** Cell counting kit was used to detect viability of SKOV3; **D** Cell counting kit was used to detect viability of A2780. **E** Transwell detected cell migration; **F** Transwell assessed the cell invasion. ^*^P < 0.05, ^**^P < 0.01, ^***^P < 0.001 *vs.* Control

RT-qPCR results revealed that in SKOV3 and A2780 cells, the CENPM expression level in the si-NC group suggested no significant difference, compared with the control group, while the CENPM expression levels in the si-CENPM-1, si-CENPM-2, and si-CENPM-3 groups were significantly decreased (Fig. 5A). Among them, si-CENPM-1 had the highest transfection efficiency. Western blot was performed and showed the same results as RT-qPCR detection, further confirming the RT-qPCR results (Fig. 5B). Therefore, the subsequent experiments were conducted using si-CENPM-1.

CCK-8 results suggested that in SKOV3 and A2780 cells, compared to the control group, there were no significant changes in si-NC group, while cell proliferation of SKOV3 and A2780 cells in the si-CENPM group was significantly suppressed (*P* < 0.001), indicating that knocking down CENPM significantly inhibited the proliferation ability of OC cells (Fig. 5C-D). Additionally, in SKOV3 and A2780 cells, the cell migration, and invasion abilities in the si-CENPM group were notably decreased compared with the control group (Fig. 5E-F).

### Knockdown of CENPM promotes pyroptosis in OC cells

**Fig. 6:**
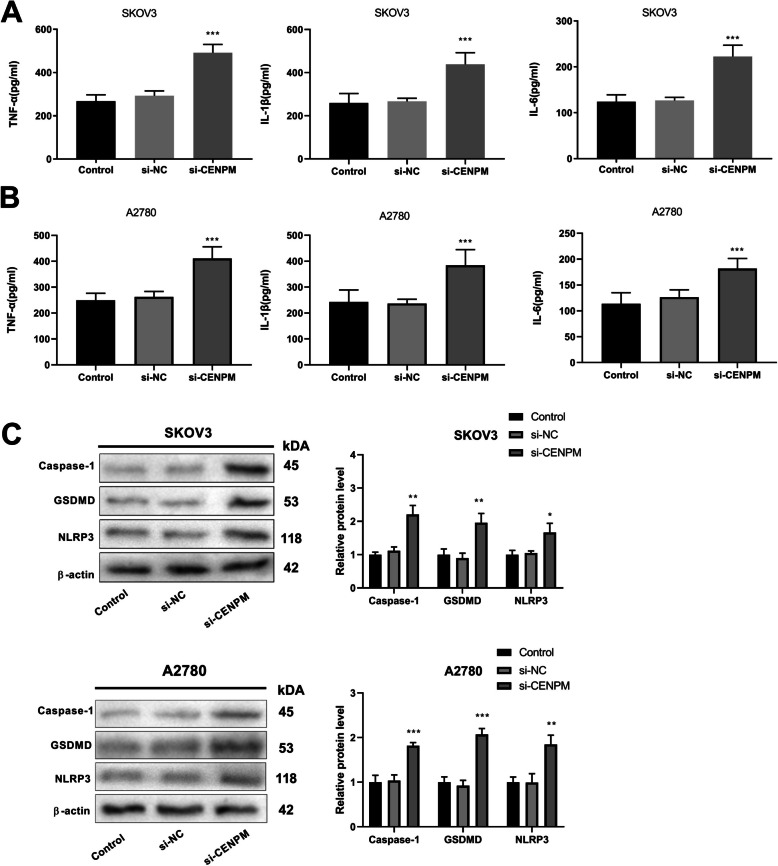
Effect of CENPM knockdown on pyroptosis. **A** The level of inflammatory factors in SKOV3 cells; **B** The level of inflammatory factors in A2780 cells; **C** Western blot was used to detect the expression of pyroptosis-related proteins in SKOV3 and A2780 cells. ^*^P < 0.05, ^**^P < 0.01, ^***^P < 0.001 *vs*. Control

Compared to the control group, the concentrations of TNF-α, IL-1β, and IL-6 in the si-CENPM group were significantly increased (Fig. 6A-B). Western blot assay demonstrated that in SKOV3 and A2780 cells, compared with the control group, the expression levels of pyroptosis-related proteins in the si-CENPM group were significantly increased (Fig. 6C).

### Knockdown of CENPM inhibits tumor growth, metastasis, and pyroptosis in mice

**Fig. 7:**
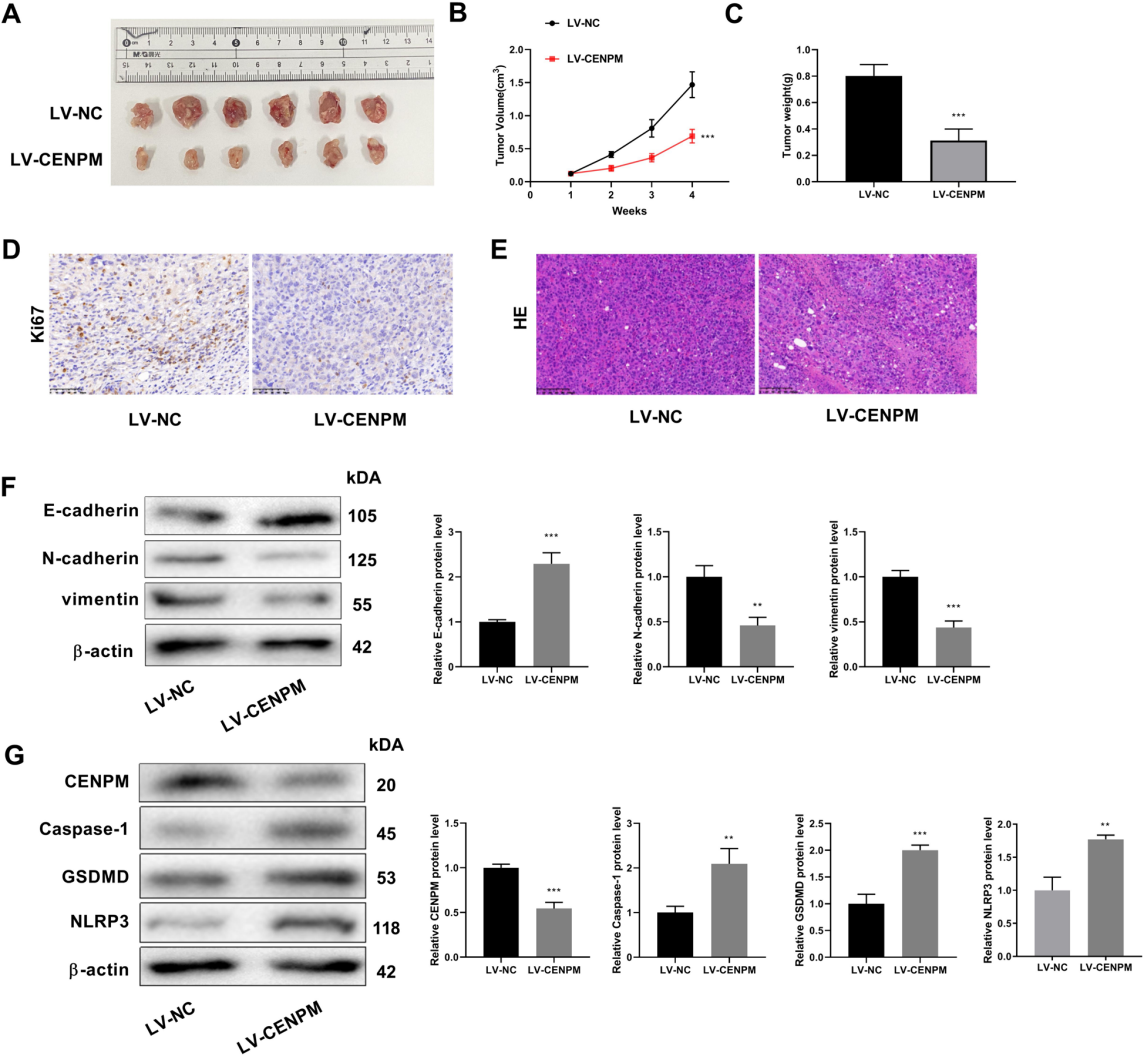
Effect of CENPM knockdown on tumor. **A** Subcutaneous tumor in each group; **B** Growth curve of subcutaneous tumor in mice; **C** Weight of subcutaneous tumor in mice; **D** Immunohistochemical analysis detected Ki67 levels; **E** Hematoxylin & eosin staining of subcutaneous tumor (Amplification: 200 × , Scale: 100 μm); **F** Western blot detected epithelial-mesenchymal transition-related proteins; **G** Western blot to detect the expression of CENPM and pyroptosis-related proteins in tumor tissues. ^**^P < 0.01 ^***^P < 0.001 *vs*. LV-NC group

The mice in the LV-CENPM group presented significantly lower tumor volumes and weights than those in LV-NC group (Fig. 7A-C). Immunohistochemical staining showed that Ki67 expression level was decreased in LV-CENPM group (Fig. 7D). HE staining suggested that knockdown of CENPM inhibited malignancy of OC (Fig. 7E).

Epithelial-mesenchymal transition-related proteins were detected to evaluate tumor metastasis by western blot assay (Fig. 7F). The results showed that knockdown of CENPM increased the protein expression level of E-cadherin, while decreasing the protein expression level of N-cadherin and vimentin. Western blot results demonstrated that the expression level of CENPM protein in tumor tissues of mice was significantly reduced after CENPM knockdown and the expression levels of pyroptosis-related proteins were significantly increased (Fig. 7G). These results further confirmed the inhibitory effect of CENPM knockdown on tumor progression and pyroptosis.

### Knockdown of CENPM suppresses the development of OC via activating cGAS-STING pathway

**Fig. 8:**
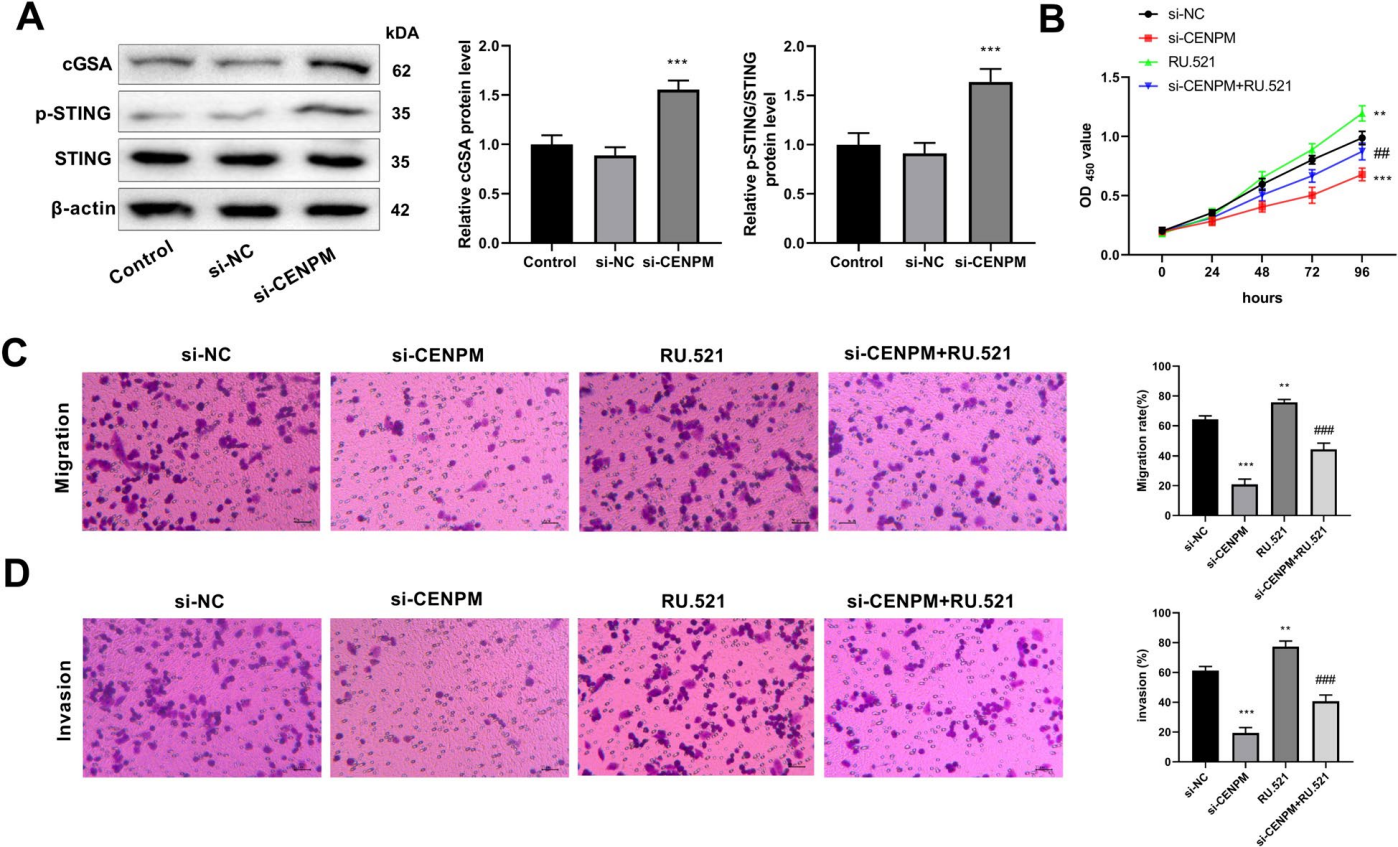
Effect of CENPM knockdown on cGAS-STING pathway. **A** Western blot was used to detect the protein expression levels of pathway proteins; **B** Cell counting kit was used to detect cell viability. **C** Transwell detected cell migration; **D** Transwell assessed the cell invasion. ^**^P < 0.01, ^***^P < 0.001 *vs.* Control; ^##^*P* < 0.01, ^###^*P* < 0.001 *vs.* si-CENPM

Western blot results revealed that the expression levels of pathway proteins, cGAS, STING, and p-STING in cGAS-STING pathway were significantly higher in si-CENPM group than those in si-NC group (Fig. 8A). Moreover, the treatment of RU.52 reversed the inhibitory effect of CENPM knockdown on proliferation, migration, and invasion of OC cells (Fig. 8B-D).

## Discussion

OC is a heterogeneous disease with the greatest fatality rate and the worst prognosis among gynecological malignancies [34]. Therefore, it is critical to develop new biomarkers for screening in the general population, particularly in high-risk populations such as those with a family history of cancer [35]. Pyroptosis is a non-apoptotic death that has shown great potential in cancer prevention and treatment [36]. In this investigation, we screened and identified the hub genes for OC based on PPI network of DEGs related to OC. The hub genes we selected had highly predictive value for the diagnosis of OC. After literature research, CENPM, a novel gene that has rarely been reported in OC, was screened out as a promising biomarker of OC. CENPM was highly expressed in OC cells. After CENPM knockdown, the migration, invasion, and viability of OC cells, as well as the growth and metastasis of tumors in mice were inhibited, indicating that CENPM silencing inhibited the development of OC. We focused on the effect of pyroptosis on OC development. CENPM knockdown promoted pyroptosis in vivo and in vitro. Further study found that knockdown of CENPM activated cGAS-STING pathway and the cGAS-STING pathway inhibitor reversed the inhibitory effect of CENPM knockdown on OC. Therefore, we concluded that knockdown of CENPM inhibited OC progression by promoting pyroptosis and activating cGAS-STING pathway.

Biomarkers play crucial roles in cancers, and an increasing number of prognostic markers for OC have been identified, making treatment of OC more effective and precise [37]. In this study, we identified KIFC1, PCLAF, CDCA5, KNTC1, MCM3, OIP5, CENPM, KIF15, and ASF1B as hub genes with high prediction value for OC based on GSE12470 and GSE16709 datasets. KIFC1 promotes cell proliferation, migration, and epithelial-mesenchymal transition through interaction with CENPE in OC and may be a potential biomarker and therapeutic target for patients with OC [38]. PCLAF silencing inhibits the proliferation and promotes apoptosis of OC cell lines, which can be used as a target for OC therapy [39]. Based on GSE27651, GSE38666, GSE40595, and GSE66957 datasets, overexpression of CDCA5 in patients with epithelial OC is associated with poor prognosis in patients with epithelial OC, which is one of the hub genes of epithelial OC [40]. Tissue microarray analysis shows that the expression of KNTC1 in vivo is higher in high-grade squamous intraepithelial lesions than in normal cervix, and KNTC1 is a novel tumor suppressor that can prevent the occurrence and development of cervical cancer [41]. MCM3 is a favorable prognostic marker for salpingo-ovarian high-grade serous carcinoma, and high MCM3 protein levels are also associated with significantly prolonged disease-specific survival [42]. Sangerbox online analysis tool is used to analyze the expression of OIP5 in OC and various human tumors, and OIP5 is highly expressed in OC based on the analysis of GSE12470, GSE14407 and GSE54388 datasets, which may be an important biomarker for the prognosis and diagnosis of OC [43]. By comparative gene expression analysis, CENPM is identified as one of key candidate genes for epithelial OC [44]. Based on the analysis of 91 OC samples and 22 normal ovarian tissues from GSE18520, GSE54388, and GSE27651 gene expression profiles, KIF15 has promising predictive value for the development and prognosis of OC and can be used as a candidate target for the diagnosis and treatment of OC [45]. To construct gene interaction networks based on weighted gene co-expression network analysis, using pancreatic cancer expression profiles from the Cancer Genome Atlas, ASF1B is a promising regulator for pancreatic cancer detection and treatment [46]. All in all, KIFC1, PCLAF, CDCA5, KNTC1, MCM3, OIP5, CENPM, KIF15, and ASF1B are hub genes of OC, which had good specificity and sensitivity in the diagnosis of OC.

Centromere dysfunction, which results in changes in chromosome number, is thought to be closely associated with human cancers [47]. So, as a member of centromeric protein family, CENPM plays a crucial role in progression of various tumors. CENPM is up-regulated in lung adenocarcinoma, which can promote lung adenocarcinoma cell cycle, cell proliferation, migration, and invasion, while inhibiting apoptosis [48]. In pancreatic cancer, CENPM is overexpressed, and down-regulation of CENPM inhibits pancreatic cancer cell proliferation, alters cell cycle, and restricts migration, and invasion [49]. The expression of CENPM is up-regulated in liver cancer, with poor prognosis and silencing CENPM suppresses cell proliferation, migration, and invasion, while depletion of CENPM can promote apoptosis and stagnant cell cycle [13]. We found that CENPM was significantly up-regulated in OC and knockdown of CENPM significantly inhibits proliferation, migration, invasion, and tumor growth of OC. Taken together, knockdown of CENPM suppressed the progression of OC.

Research suggests a link between pyroptosis and OC [50]. Many biomarkers have been implicated in the pyroptosis of OC cells. A total of 31 DEGs that may affect pyroptosis are screened out between OC and normal ovarian tissue, and 7 of them are identified to be involved in tumor immunity and prognosis prediction of OC, including 3 down-regulated genes (PLCG1, ELANE, PJVK) and 4 up-regulated genes (AIM2, CASP3, CASP6, and GSDMA) [51]. The expression of growth-station-specific transcription 5 of lncRNA is inhibited in OC tissues and can cause the development of inflammatory bodies, inducing pyroptosis in vivo and in vitro [52]. In addition, lncRNA HOTTIP is up-regulated, and its deletion can lead to pyroptosis and hinder the progress of OC [53]. It has been reported that α-Neta directly induces pyroptosis of epithelial OC cells by activating Caspase-4 cleavage of GSDMD, thereby inhibiting their proliferation [54]. In the present study, we illustrated that knockdown of CENPM caused an increase in the expression of pro-inflammatory factors and pyroptosis-related proteins in OC cells and tumors. In brief, knockdown of CENPM promoted pyroptosis to inhibit the development of OC.

Some OC cells suggested unbalanced STING pathway signals by inhibiting STING and its upstream sensor cGAS [55]. Niraparib combined with Entinostat can enhance the cytotoxicity of immune cells by activating the cGAS-STING pathway and promoting the infiltration of pre-immune cells into ascites, thus inhibiting the growth of OC [56]. CX-5461 can make STING transcriptional upregulations mediated by cGAS, thus activating the innate immune pathway and improving the efficacy of epithelial OC immunotherapy [57]. In high-grade serous OC, cGAS/ STING-mediated activation of the restrictive aging-related secretory phenotype is sufficient to induce immune infiltration and sensitize the homologous recombination deficient high-grade serous OC to immune checkpoint blockade [58]. Additionally, the cGAS/STING pathway plays an important role in the disease by regulating pyroptosis. 4-octyl itaconate can ameliorate alveolar macrophage pyroptosis in acute respiratory distress syndrome by rescuing mitochondrial dysfunction and inhibiting the cGAS/STING pathway [59]. Activation of the cGAS-STING axis contributes to neuroinflammation and induces inflammasome activation and microglial pyroptosis in a mouse model of neuroinflammatory injury and cerebral venous sinus thrombosis [60]. In our research, knockdown of CENPM activated cGAS-STING pathway and the cGAS inhibitor reversed the inhibitory effect of CENPM knockdown on OC progression. Taken together, knockdown of CENPM inhibited OC progression via activating cGAS-STING pathway and promoting pyroptosis of OC cells. The cGAS/STING pathway is a promising target for the treatment of OC.

This research also has some limitations. For instance, due to the limited funding and insufficient experimental period, we did not use two or more siRNAs to knock down CENPM to exclude off-target effects. This study also lacks relevant validations based on clinical samples due to the limitations of laboratory conditions and clinical samples. Additionally, the exploration of the role of chromosomal segregation defect caused by CENPM deficiency will contribute to improving our findings. We will improve the laboratory conditions and collect more clinical samples to refine our research in the future.

In conclusion, KIFC1, PCLAF, CDCA5, KNTC1, MCM3, OIP5, CENPM, KIF15, and ASF1B were identified as hub genes with high diagnostic value. CENPM, a key hub gene of OC, was overexpressed in OC, which was determined as a potential biomarker. Knockdown of CENPM promoted pyroptosis and inhibited the progression of OC via activating cGAS-STING pathway. Our findings indicate that CENPM may be a promising molecular target and a potential therapeutic for OC, which can provide a new insight into pathogenesis of OC and help us understand the underlying mechanism associated with OC.

### Supplementary Information

**Supplementary Material 1.**

**Supplementary Material 2.**

## Data Availability

The datasets generated and/or analyzed during the current study are available in GEO of National Center for Biotechnology Information repository, [www.ncbi.nlm.nih.gov/geo/browse/?view=series]. The original analysis data, volcano graph, and data chart can be obtained from GEO2R repository, [www.ncbi.nlm.nih.gov/geo/geo2r]. The data of hub genes expression in tumor tissue and normal tissue can be downloaded from GEPIA2 repository, [http://gepia2.cancer-pku.cn/#index]. Primer sequences are available from National Center for Biotechnology Information repository, [https://www.ncbi.nlm.nih.gov/].
