# Supplementary material for: Knockdown of CENPM activates cGAS-STING pathway to inhibit ovarian cancer by promoting pyroptosis
Source: BMC Cancer. 2024 May 1;24:551. doi: 10.1186/s12885-024-12296-5 (PMC11064423; doi:10.1186/s12885-024-12296-5)
Supplement: Supplementary file 1 — Supplementary Material 1. [file 12885_2024_12296_MOESM1_ESM.docx]

Table S1 Sequence information of si-RNA and primers

| Name | Sequence (5’-3’) |
| --- | --- |
| si-NC-F | UUCUCCGAACGUGUCACGUTT |
| si-NC-R | ACGUGACACGUUCGGAGAATT |
| si-CENPM-1-F | GGUUAAUCUUCACAGCAAAUA |
| si-CENPM-1-R | UUUGCUGUGAAGAUUAACCAC |
| si-CENPM-2-F | GCAAAUACAGUCUCCAGAACA |
| si-CENPM-2-R | UUCUGGAGACUGUAUUUGCUG |
| si-CENPM-3-F | UGAUCGUGUUUGUGGUUAAUC |
| si-CENPM-3-R | UUAACCACAAACACGAUCAGG |
| GAPDH-F | GAGTCAACGGATTTGGTCGT |
| GAPDH-R | TTGATTTTGGAGGGATCTCG |
| CENPM-F | CTTTGCCCTCCAGTGTGAAT |
| CENPM-R | GAAACACACCTTCCCCAAGA |
| KIFC1-F | AGCCTGAGAAGAAACGGACA |
| KIFC1-R | GAACAGCAGGAACTGGCTTC |
| PCLAF -F | ACAGAAAAGTGGTGGCTGCT |
| PCLAF -R | CCTGCCTCTTCAGGAATCTG |
| CDCA5-F | GCTGAGGGTTGAGGTGAGAG |
| CDCA5-R | GAAACCCACGGAACTGAAAA |
| KNTC1-F | TGCAGCTCAAAGTCCACATC |
| KNTC1-R | GTCCATTTCCAGGTGCTGTT |
| MCM3 -F | TCAAGCCTGTCCTGACACAG |
| MCM3 -R | CAGGTCCACAGTCTTGCTCA |
| OIP5-F | CGCACAATCGCTTAAAATCA |
| OIP5-R | CATTTCCCCTCCATTCTTGA |
| KIF15-F | TCTTTGCAAAAAGCGAACCT |
| KIF15-R | ACTGGTCGGGAATGAAGTTG |
| ASF1B-F | GGTTCGAGATCAGCTTCGAG |
| ASF1B-R | CATGGTAGGTGCAGGTGATG |

Table S2 The top 15 up-regulated and down-regulated genes selected from GSE12470 dataset

| **Name** | **Description** | **log2FoldChange** | **pval** | **up/down** |
| --- | --- | --- | --- | --- |
| DAPL1 | death associated protein like 1 | 7.3587606 | 2.58E-09 | up |
| KLK6 | kallikrein related peptidase 6 | 6.9229758 | 2.33E-12 | up |
| CLDN4 | claudin 4 | 6.6817457 | 1.92E-17 | up |
| KLK8 | kallikrein related peptidase 8 | 6.5815043 | 3.92E-14 | up |
| EPCAM | epithelial cell adhesion molecule | 6.5290693 | 1.14E-17 | up |
| PKP3 | plakophilin 3 | 6.4938967 | 2.20E-21 | up |
| ELF3 | E74 like ETS transcription factor 3 | 6.4767498 | 7.21E-13 | up |
| PRSS8 | protease, serine 8 | 6.298944 | 6.39E-17 | up |
| NMU | neuromedin U | 6.2919419 | 3.33E-11 | up |
| TMPRSS4 | transmembrane protease, serine 4 | 6.1968704 | 3.60E-10 | up |
| TMEM139 | transmembrane protein 139 | 6.194416 | 1.24E-19 | up |
| SCNN1A | sodium channel epithelial 1 alpha subunit | 6.1471522 | 5.32E-13 | up |
| B3GNT3 | UDP-GlcNAc:betaGal beta-1,3-N-acetylglucosaminyltransferase 3 | 6.0854521 | 1.25E-13 | up |
| PAX8 | paired box 8 | 6.0571701 | 2.54E-15 | up |
| TMEM125 | transmembrane protein 125 | 6.0368679 | 6.39E-17 | up |
| LMOD1 | leiomodin 1 | -5.2563908 | 4.05E-10 | down |
| DACT3 | dishevelled binding antagonist of beta catenin 3 | -5.2821592 | 1.15E-10 | down |
| HBA2 | hemoglobin subunit alpha 2 | -5.3343069 | 1.33E-08 | down |
| MFAP4 | microfibrillar associated protein 4 | -5.423362 | 6.13E-07 | down |
| GEM | GTP binding protein overexpressed in skeletal muscle | -5.4416063 | 7.13E-10 | down |
| MGP | matrix Gla protein | -5.8141886 | 7.74E-13 | down |
| WISP2 | WNT1 inducible signaling pathway protein 2 | -6.0247772 | 4.97E-08 | down |
| SFRP1 | secreted frizzled related protein 1 | -6.0520659 | 3.51E-10 | down |
| AOC3 | amine oxidase, copper containing 3 | -6.0530377 | 2.45E-10 | down |
| SGCA | sarcoglycan alpha | -6.0848141 | 9.75E-13 | down |
| ACTG2 | actin, gamma 2, smooth muscle, enteric | -6.5723784 | 6.06E-09 | down |
| DPT | dermatopontin | -6.7373651 | 2.68E-12 | down |
| CNN1 | calponin 1 | -6.9002331 | 6.69E-12 | down |
| PI16 | peptidase inhibitor 16 | -7.4023696 | 1.74E-13 | down |
| DES | desmin | -7.9969786 | 5.89E-12 | down |

Table S3 The top 15 up-regulated and down-regulated genes selected from GSE16709 dataset

| **Name** | **Description** | **log2FoldChange** | **pval** | **up/down** |
| --- | --- | --- | --- | --- |
| EPCAM | epithelial cell adhesion molecule | 7.55881669 | 1.93E-17 | up |
| FOLR1 | folate receptor 1 | 6.62361498 | 1.81E-11 | up |
| HLA-DRA | major histocompatibility complex, class II, DR alpha | 6.474207 | 9.42E-12 | up |
| MMP7 | matrix metallopeptidase 7 | 6.41120873 | 2.95E-07 | up |
| SCGB2A1 | secretoglobin family 2A member 1 | 6.40798298 | 4.86E-07 | up |
| CDH1 | cadherin 1 | 6.15059691 | 7.65E-14 | up |
| HLA-DMB | major histocompatibility complex, class II, DM beta | 6.00653932 | 4.54E-13 | up |
| CLDN3 | claudin 3 | 5.95623168 | 2.83E-15 | up |
| S100A4 | S100 calcium binding protein A4 | 5.79258437 | 2.11E-11 | up |
| CLDN7 | claudin 7 | 5.68704981 | 2.66E-11 | up |
| SCNN1A | sodium channel epithelial 1 alpha subunit | 5.67186427 | 1.41E-11 | up |
| KLK6 | kallikrein related peptidase 6 | 5.64428988 | 8.54E-08 | up |
| TACSTD2 | tumor-associated calcium signal transducer 2 | 5.60967636 | 7.49E-07 | up |
| FCGBP | Fc fragment of IgG binding protein | 5.59522709 | 1.31E-08 | up |
| VTCN1 | V-set domain containing T cell activation inhibitor 1 | 5.55997951 | 7.44E-06 | up |
| PTGIS | prostaglandin I2 (prostacyclin) synthase | -5.3323305 | 9.80E-11 | down |
| DKK1 | dickkopf WNT signaling pathway inhibitor 1 | -5.48718417 | 1.03E-14 | down |
| LOX | lysyl oxidase | -5.51960466 | 8.14E-12 | down |
| RGS4 | regulator of G-protein signaling 4 | -5.66521733 | 1.03E-11 | down |
| BDKRB1 | bradykinin receptor B1 | -5.75080956 | 4.63E-18 | down |
| POSTN | periostin | -6.26614121 | 1.08E-13 | down |
| IL33 | interleukin 33 | -6.29411227 | 9.67E-14 | down |
| CXCL6 | C-X-C motif chemokine ligand 6 | -6.42240802 | 2.23E-09 | down |
| SERPINB2 | serpin family B member 2 | -6.49074485 | 7.83E-11 | down |
| PAPPA | pappalysin 1 | -6.75705936 | 1.63E-16 | down |
| SERPINE1 | serpin family E member 1 | -6.83871323 | 3.06E-13 | down |
| CPA4 | carboxypeptidase A4 | -7.11844033 | 3.18E-17 | down |
| ALDH1A3 | aldehyde dehydrogenase 1 family member A3 | -7.44332727 | 1.20E-15 | down |
| ANXA10 | annexin A10 | -7.48379746 | 1.14E-18 | down |
| NPPB | natriuretic peptide B | -7.95606907 | 1.44E-23 | down |

**Figure. S1** Venn diagram of common DEGs. The numbers in each circle represents the total number of DEGs in each dataset, and the overlapping circles represent the common DEGs between comparison groups.

**
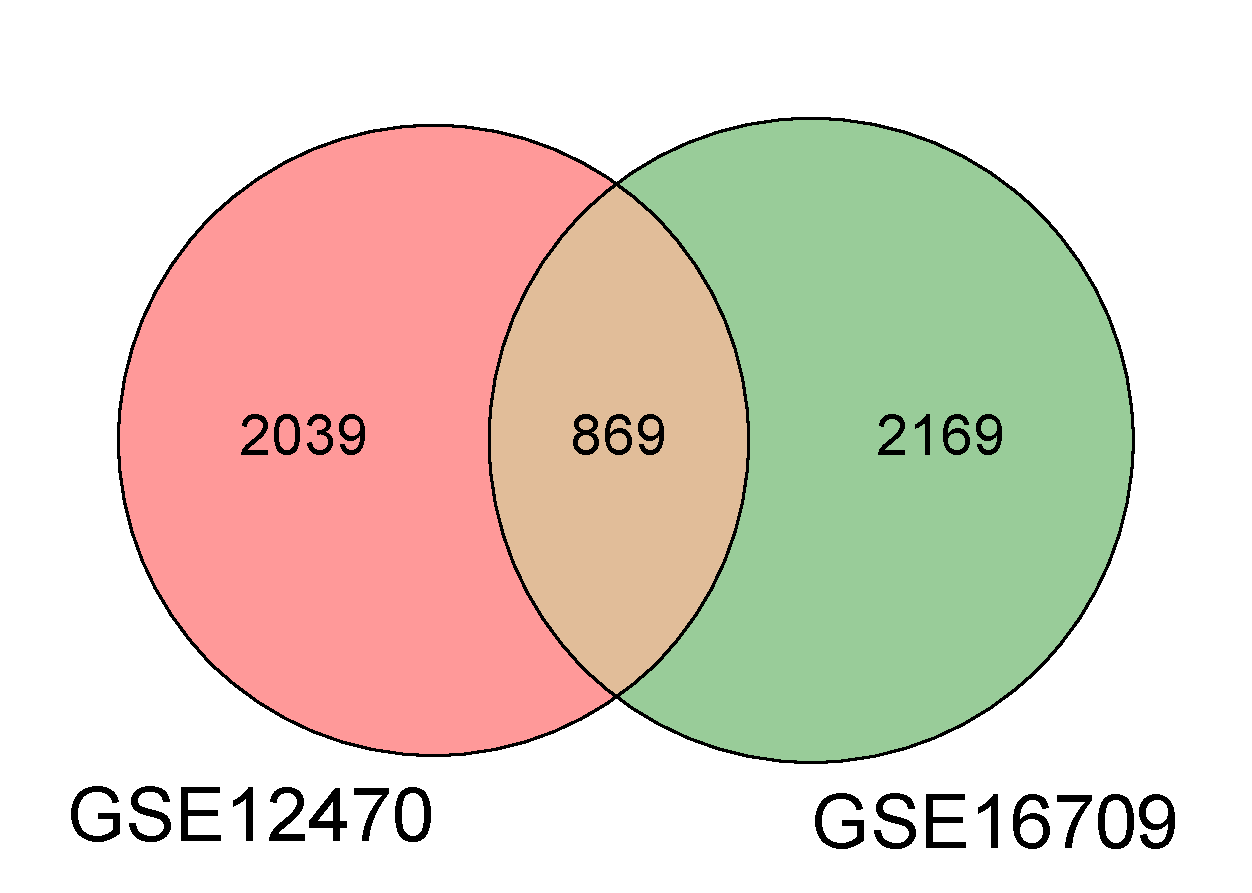
**

**Figure. S2** Analysis of common DEGs. **A.** GO enrichment analysis bubble map of DEGs; B: Histogram of Kyoto Encyclopedia of Genes and Genomes enrichment analysis of DEGs.

**
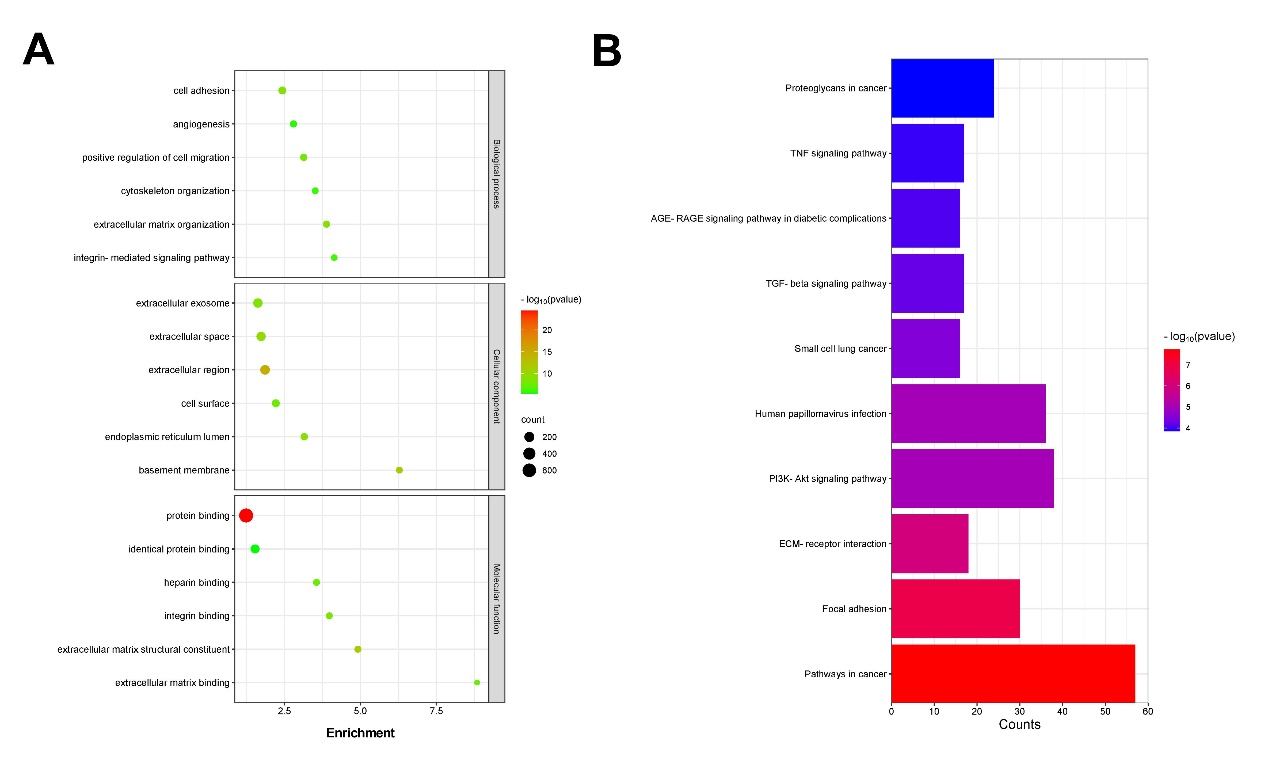
**

**Figure. S3** Protein-protein interaction network of DEGs constructed by STRING.

**
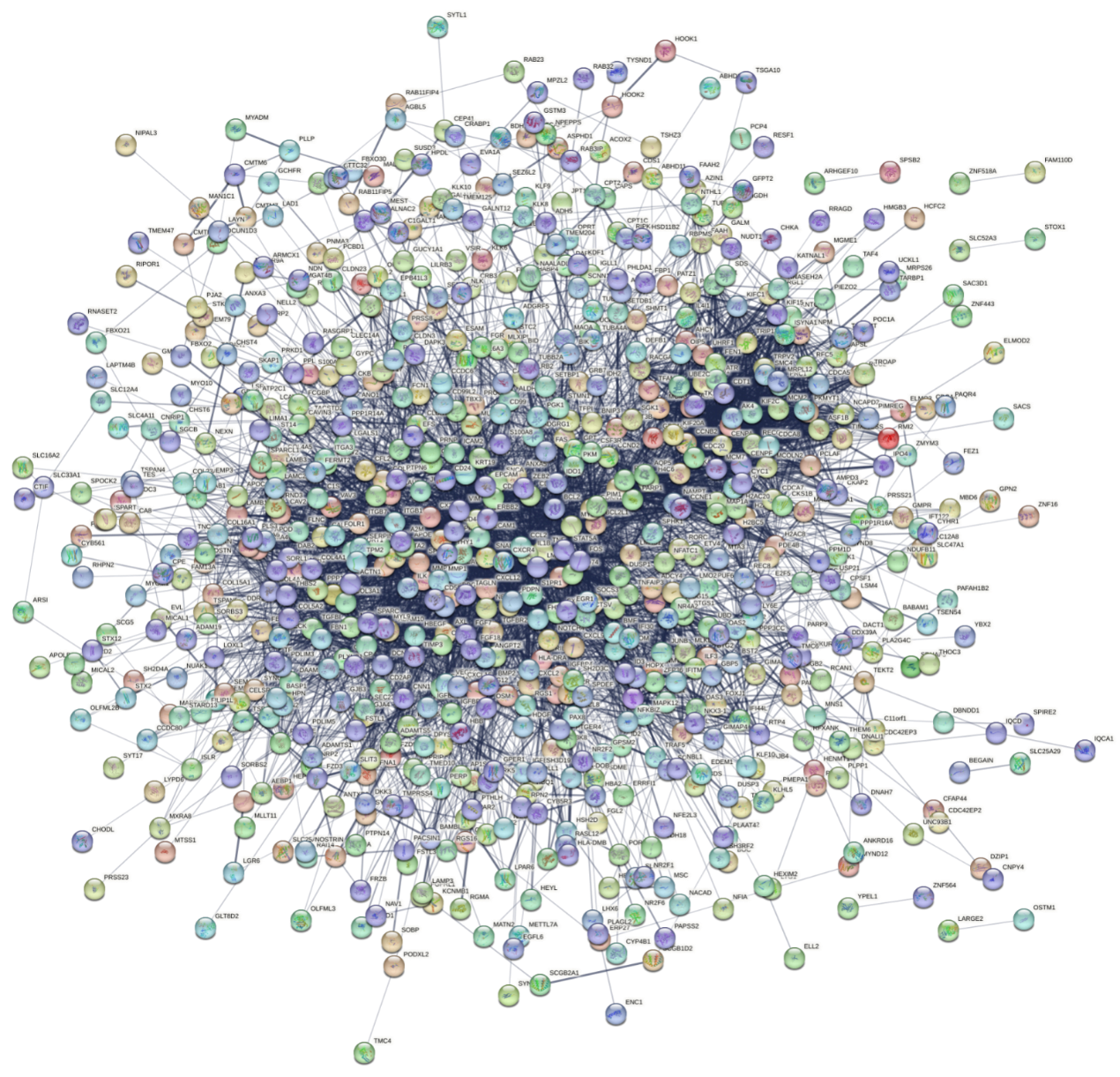
**

**Figure. S4** The identified key genes. The lines between nodes represent interactions between genes. Each node represents a gene, and an edge represents an interaction between genes.

**
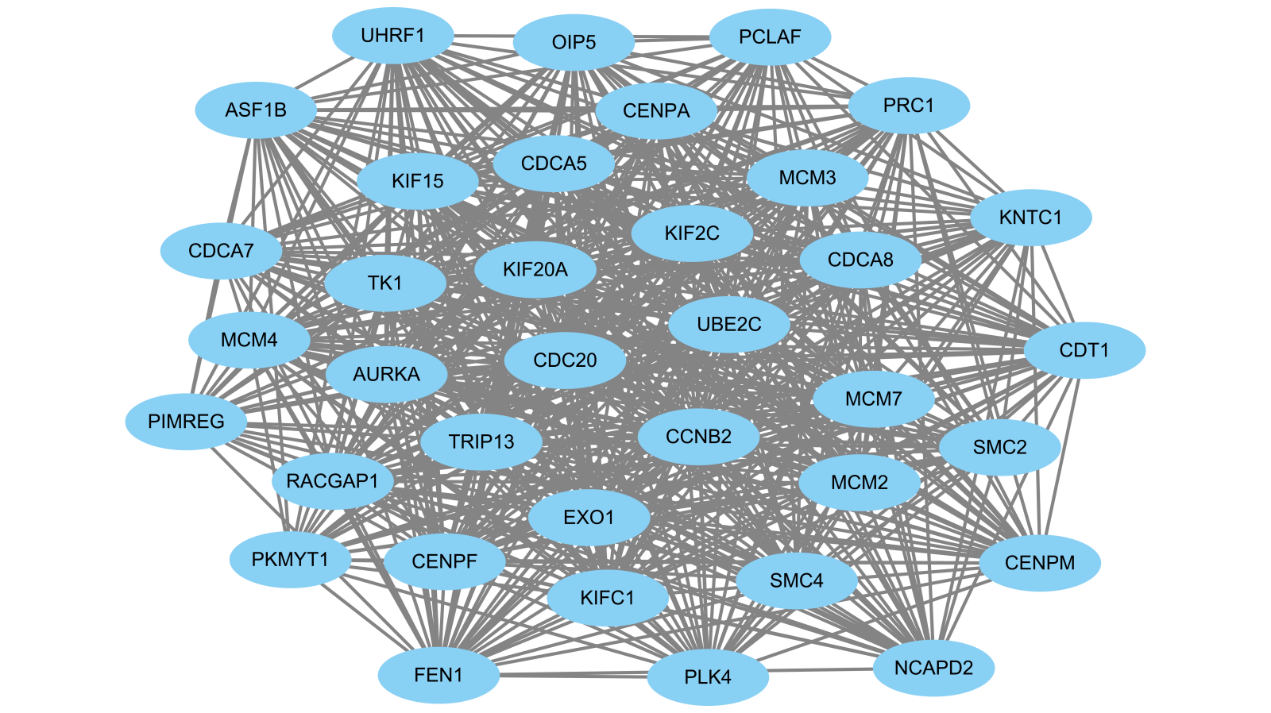
**

**Figure. S5** A: Receiver operating characteristic curve analysis of hub genes (KIFC1, PCLAF, CDCA5, KNTC1, MCM3, OIP5, CENPM, KIF15, and ASF1B) based on GSE12470 dataset.

**
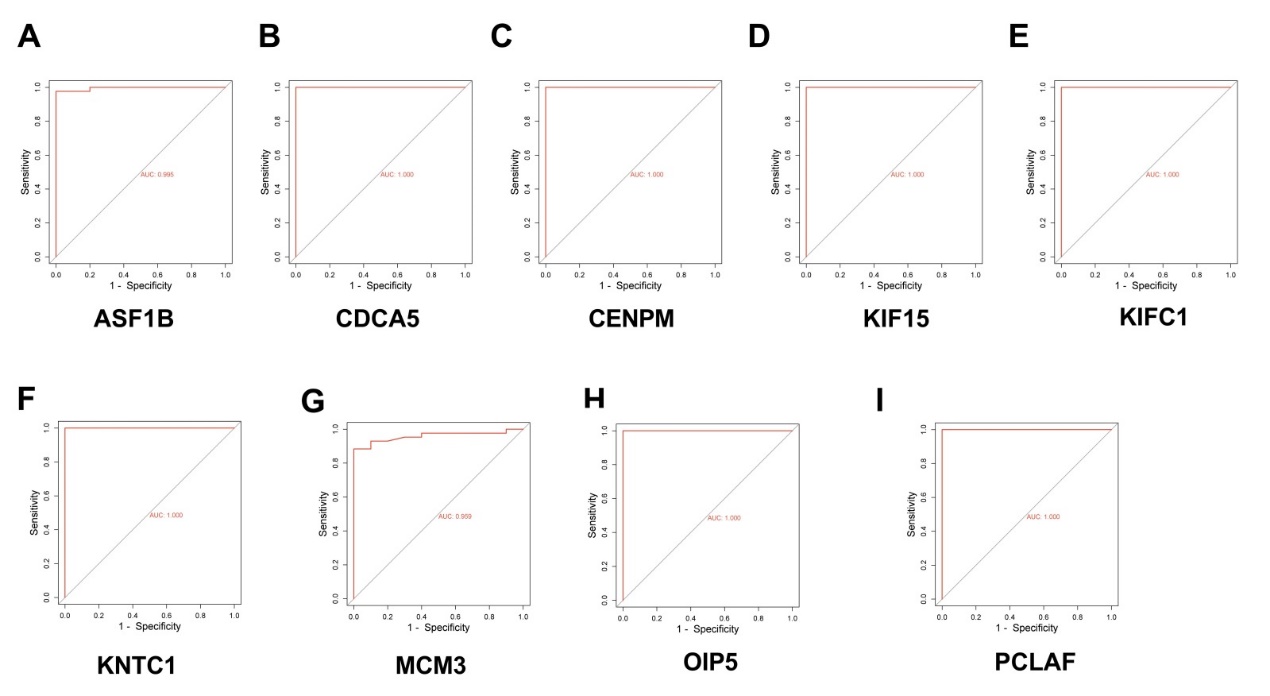
**

**Figure. S6** Receiver operating characteristic curve analysis of hub genes (KIFC1, PCLAF, CDCA5, KNTC1, MCM3, OIP5, CENPM, KIF15, and ASF1B) based on GSE16709 dataset.

**
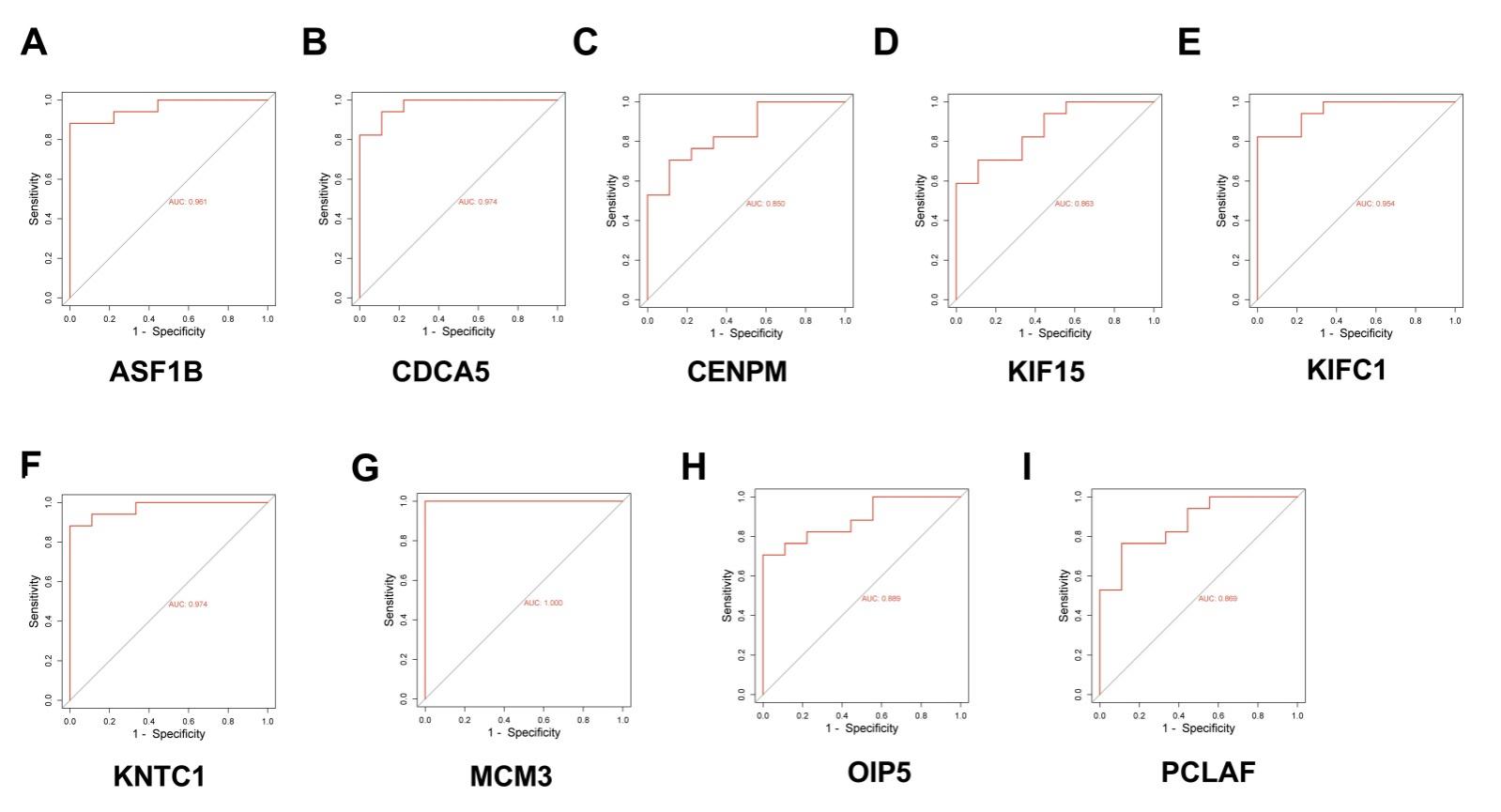
**

**Figure S7** GEPIA2 database verified the expression levels of KIFC1, PCLAF, CDCA5, KNTC1, MCM3, OIP5, CENPM, KIF15 and ASF1B in ovarian cancer.

**
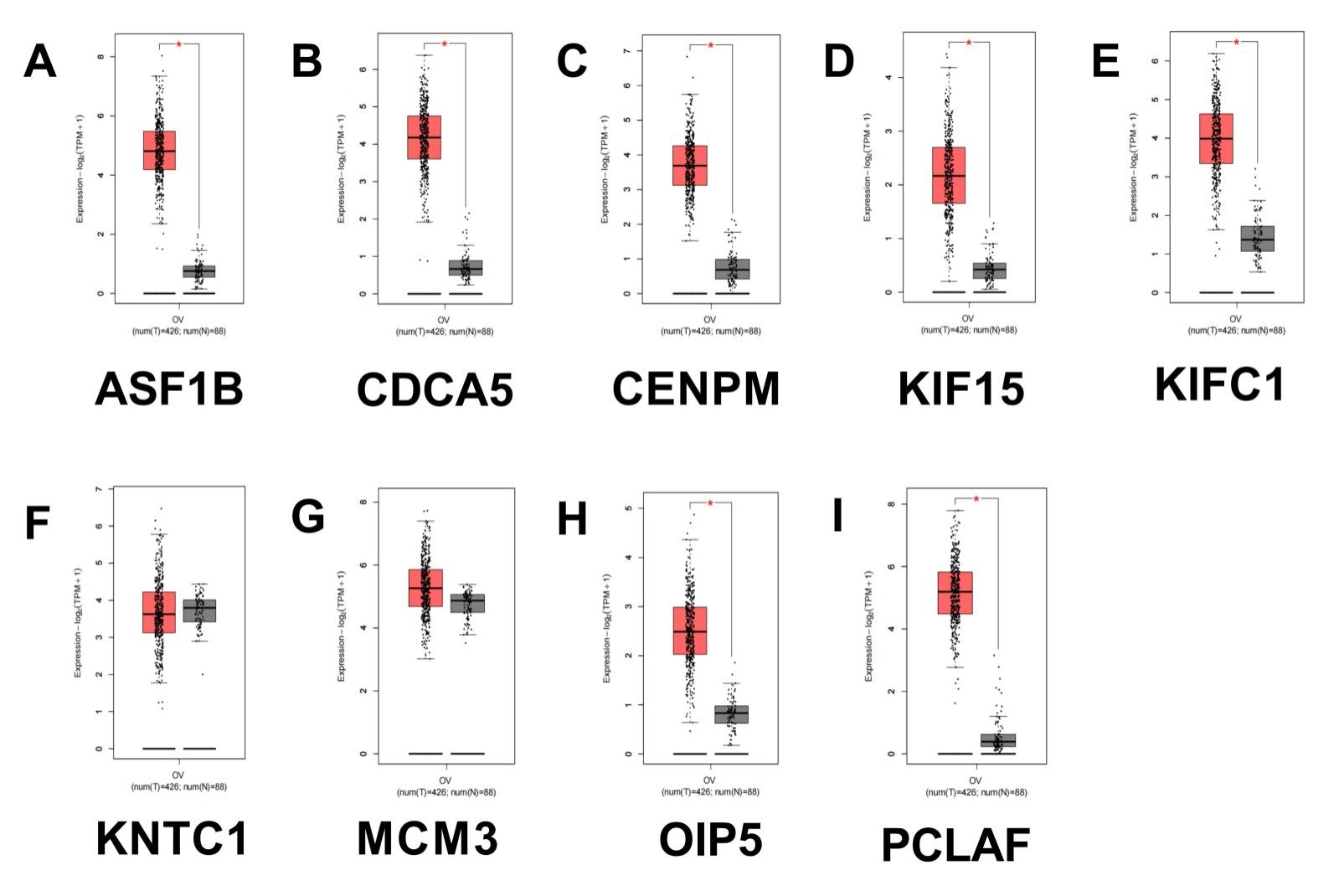
**
