# Supplementary material for: Knockdown of CENPM activates cGAS-STING pathway to inhibit ovarian cancer by promoting pyroptosis
Source: BMC Cancer. 2024 May 1;24:551. doi: 10.1186/s12885-024-12296-5 (PMC11064423; doi:10.1186/s12885-024-12296-5)

Figure 5B The CENPM protein band of SKOV3 cell

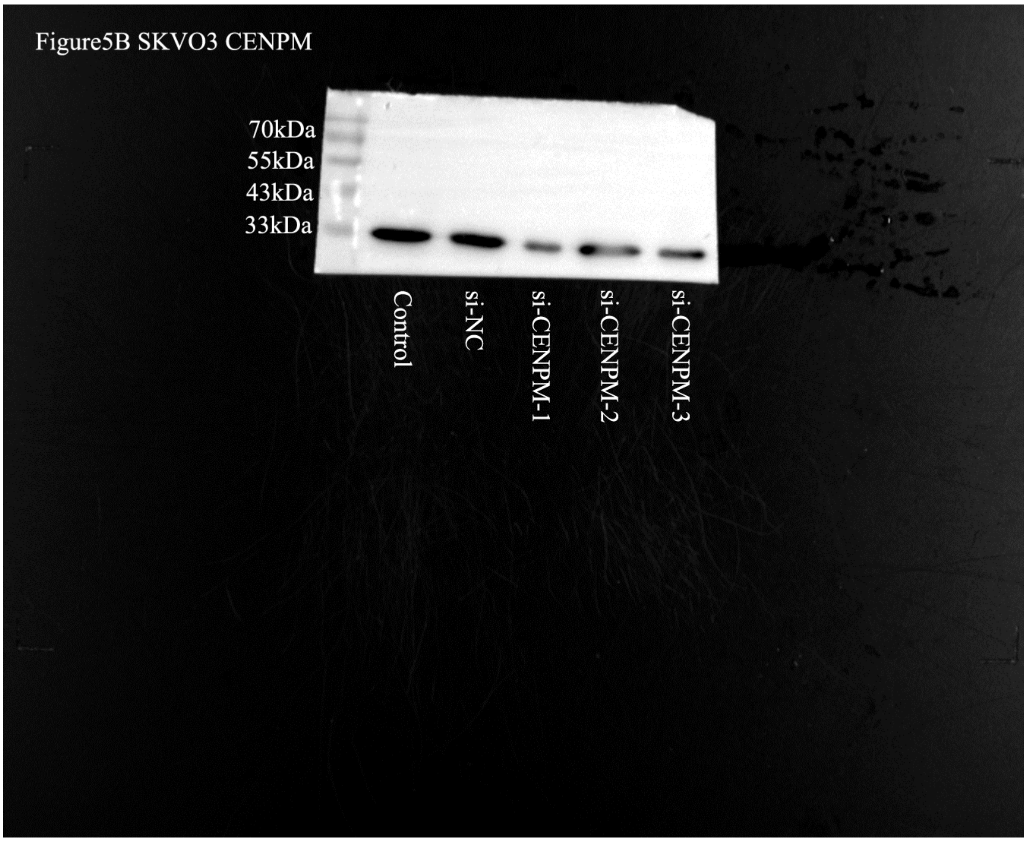

Figure 5B The β-actin protein band of SKOV3 cell

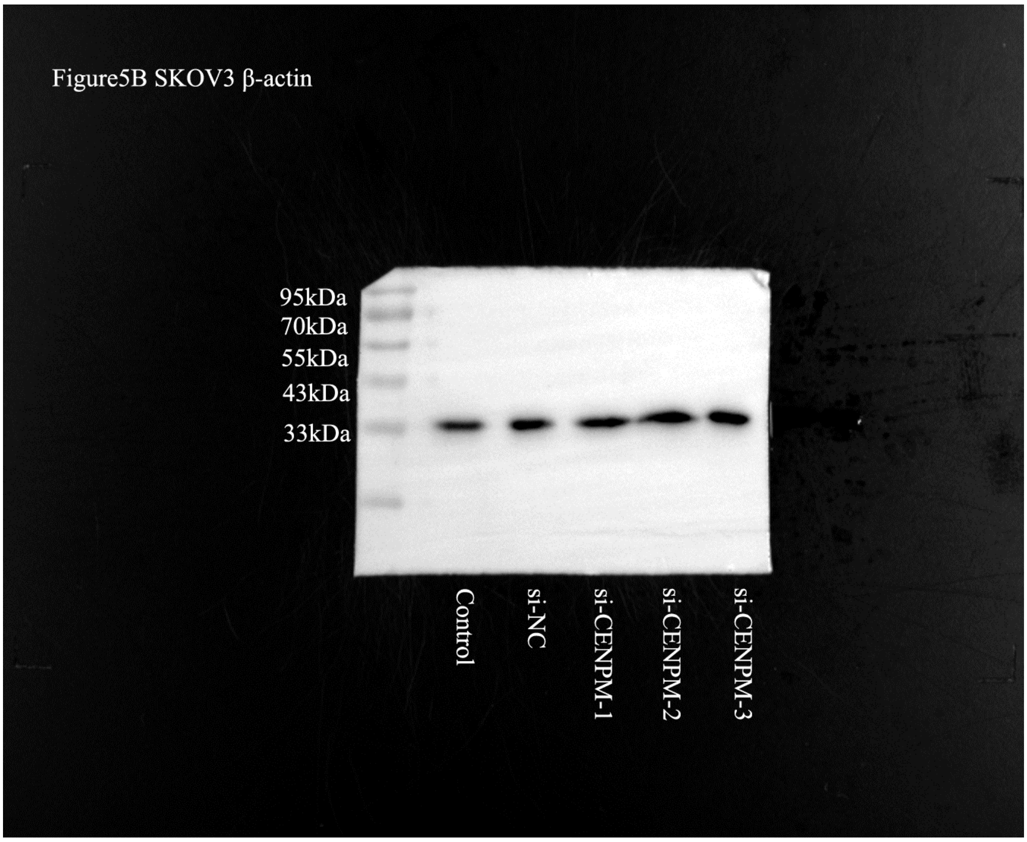

Figure 5B The CENPM protein band of A2780 cell

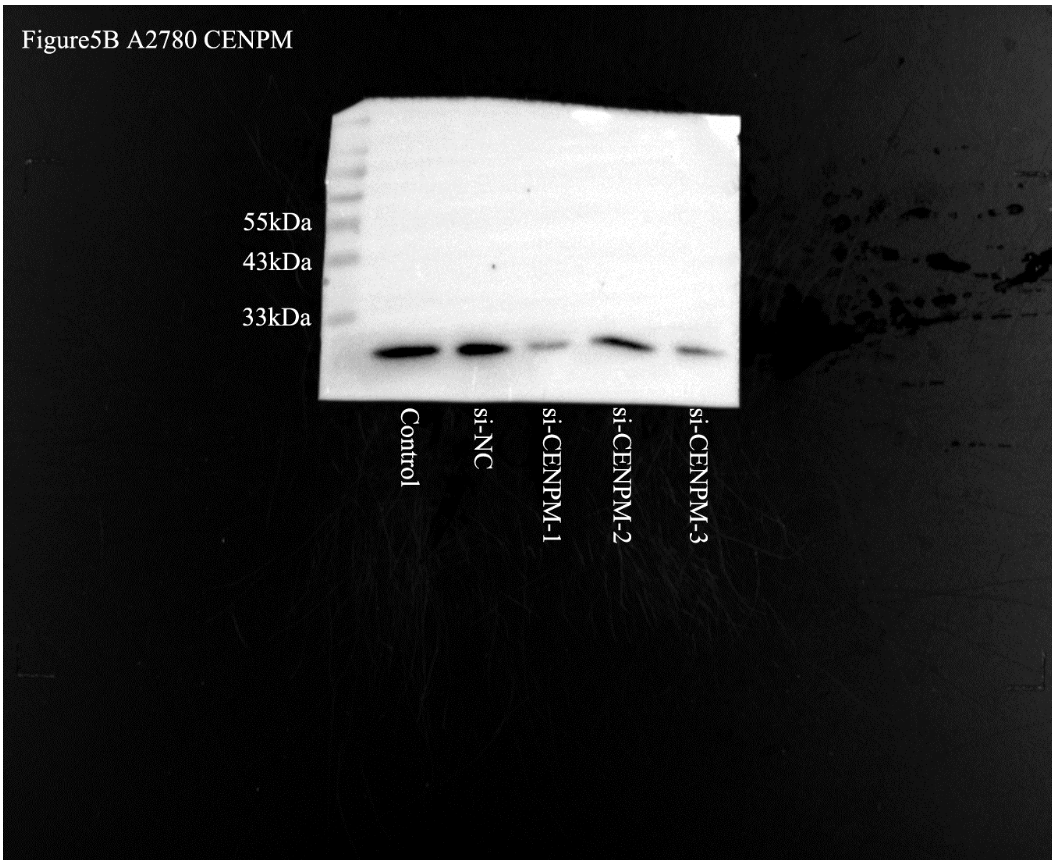

Figure 5B The β-actin protein band of A2780 cell

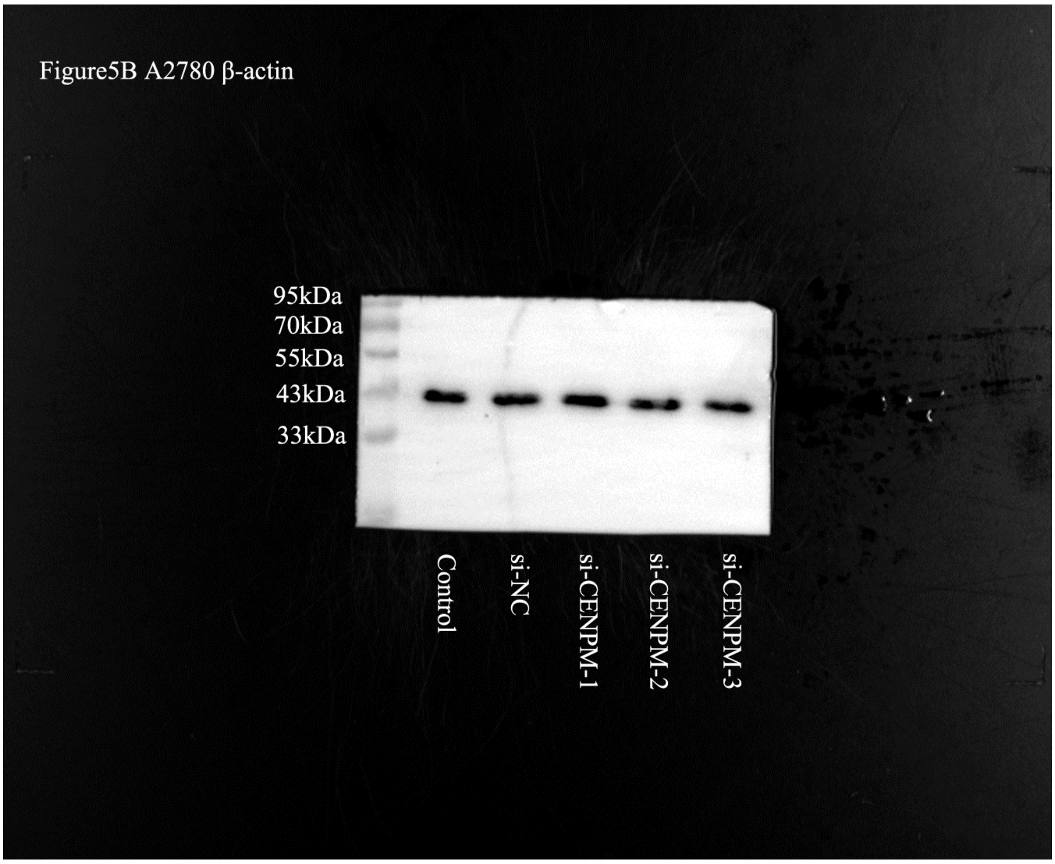

Figure 6C The Caspase-1 protein band of SKOV3 cell

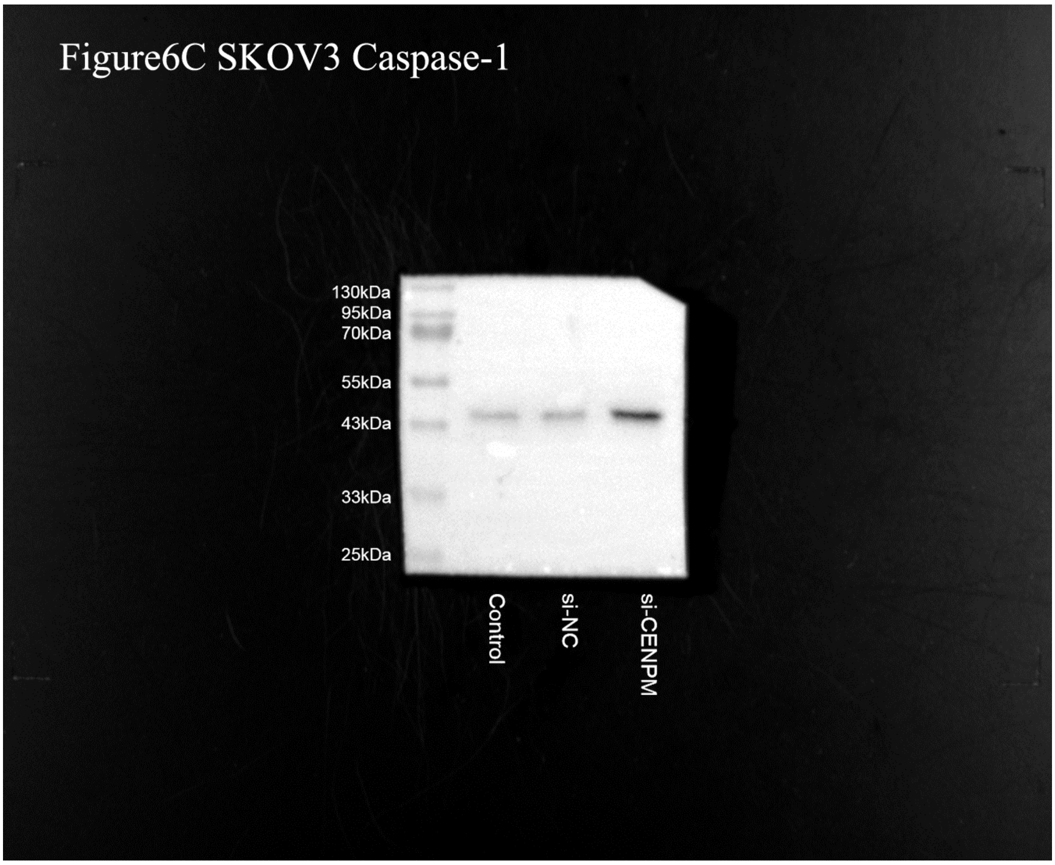

Figure 6C The GSDMD protein band of SKOV3 cell

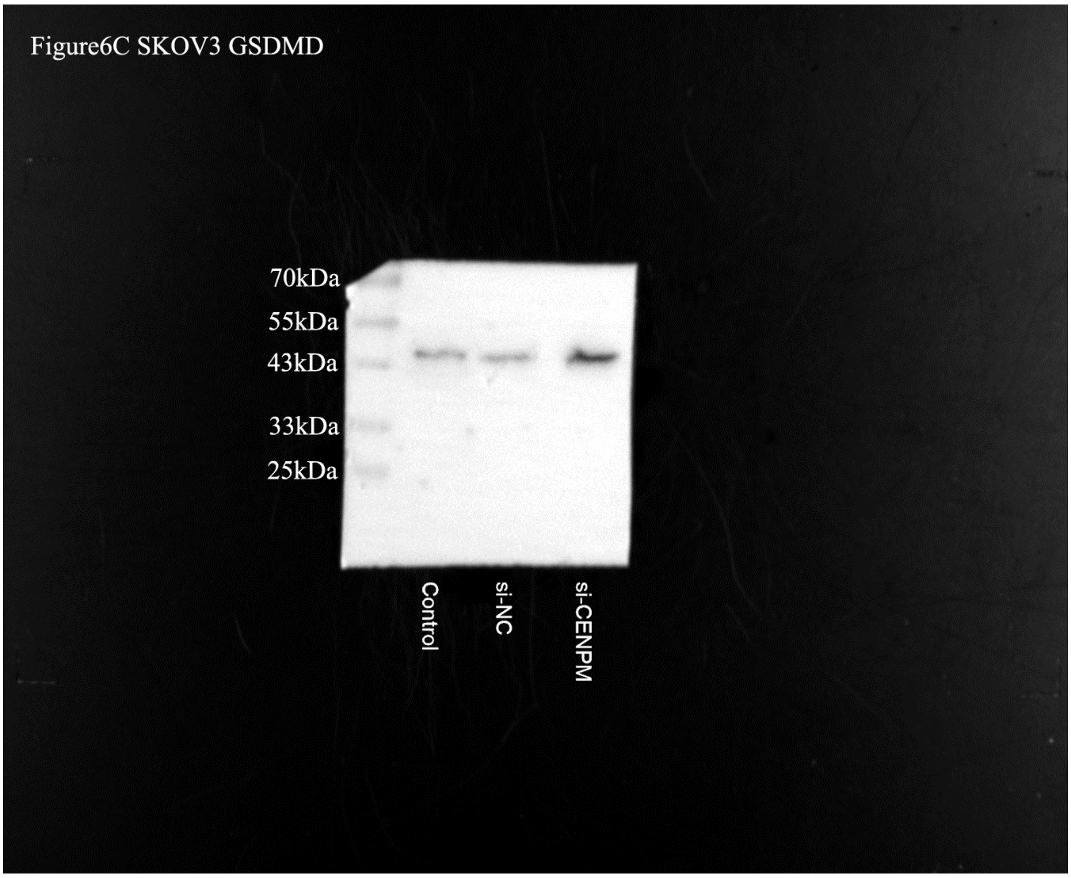

Figure 6C The NLRP3 protein band of SKOV3 cell

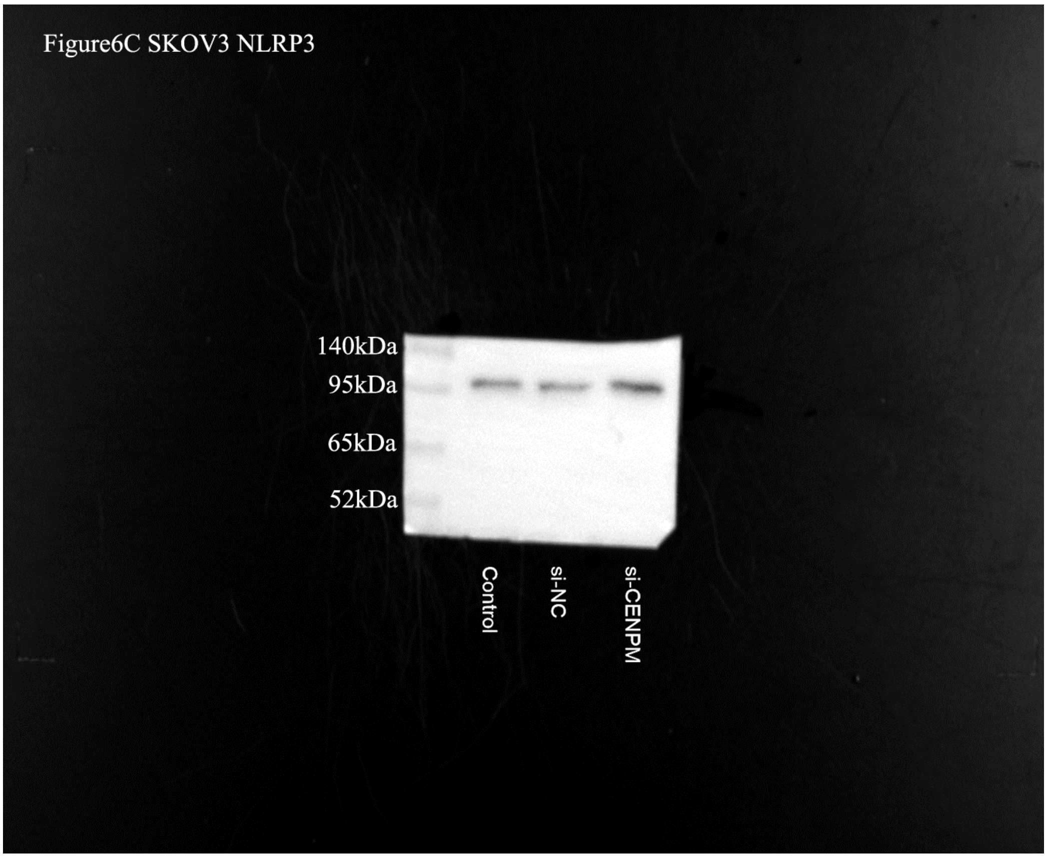

Figure 6C The β-actin protein band of SKOV3 cell

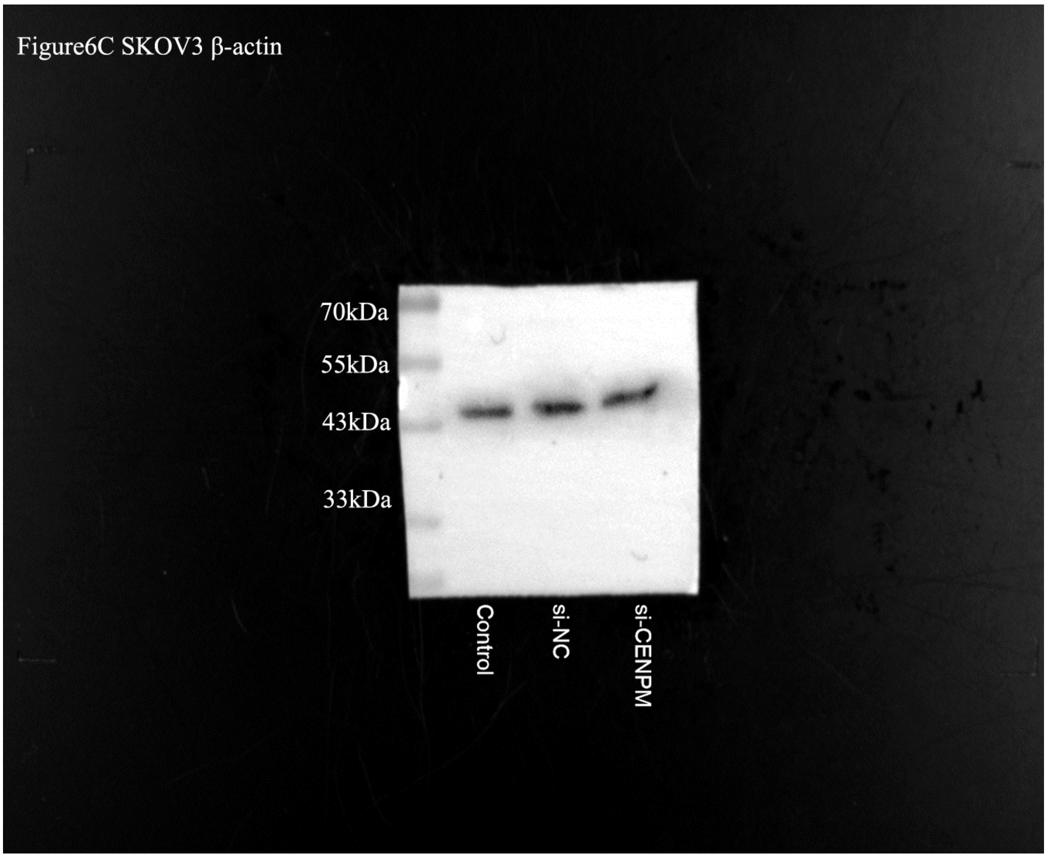

Figure 6C The Caspase-1 protein band of A2780 cell

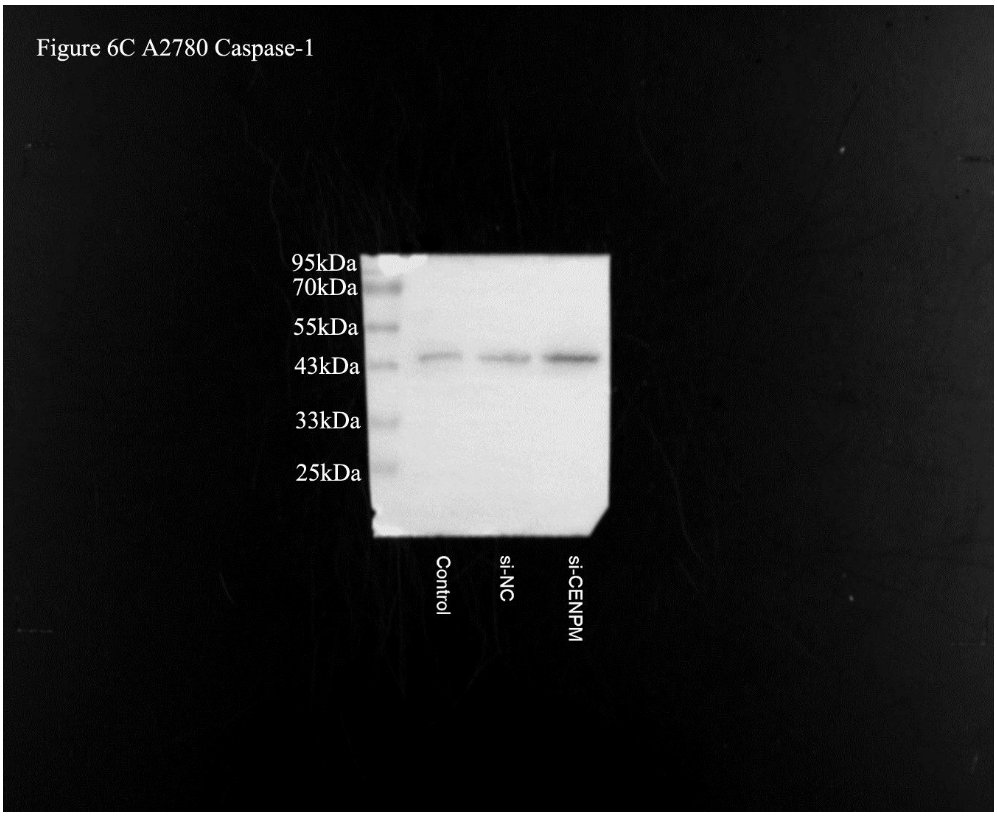

Figure 6C The GSDMD protein band of A2780 cell

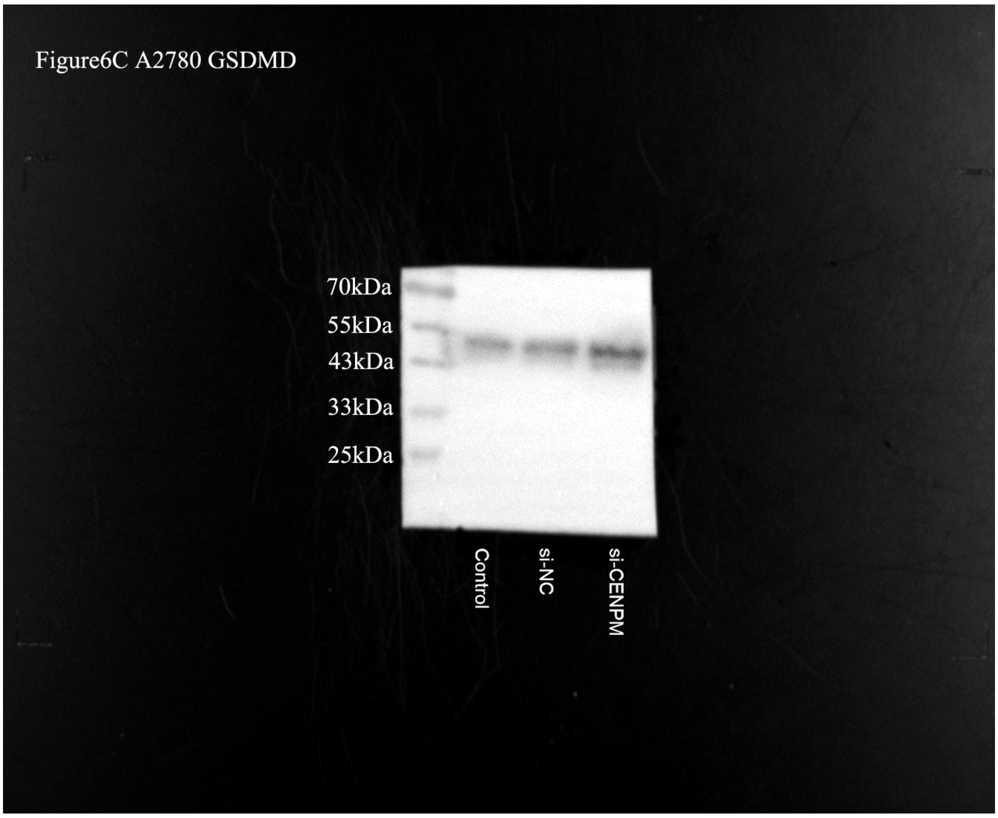

Figure 6C The NLRP3 protein band of A2780 cell

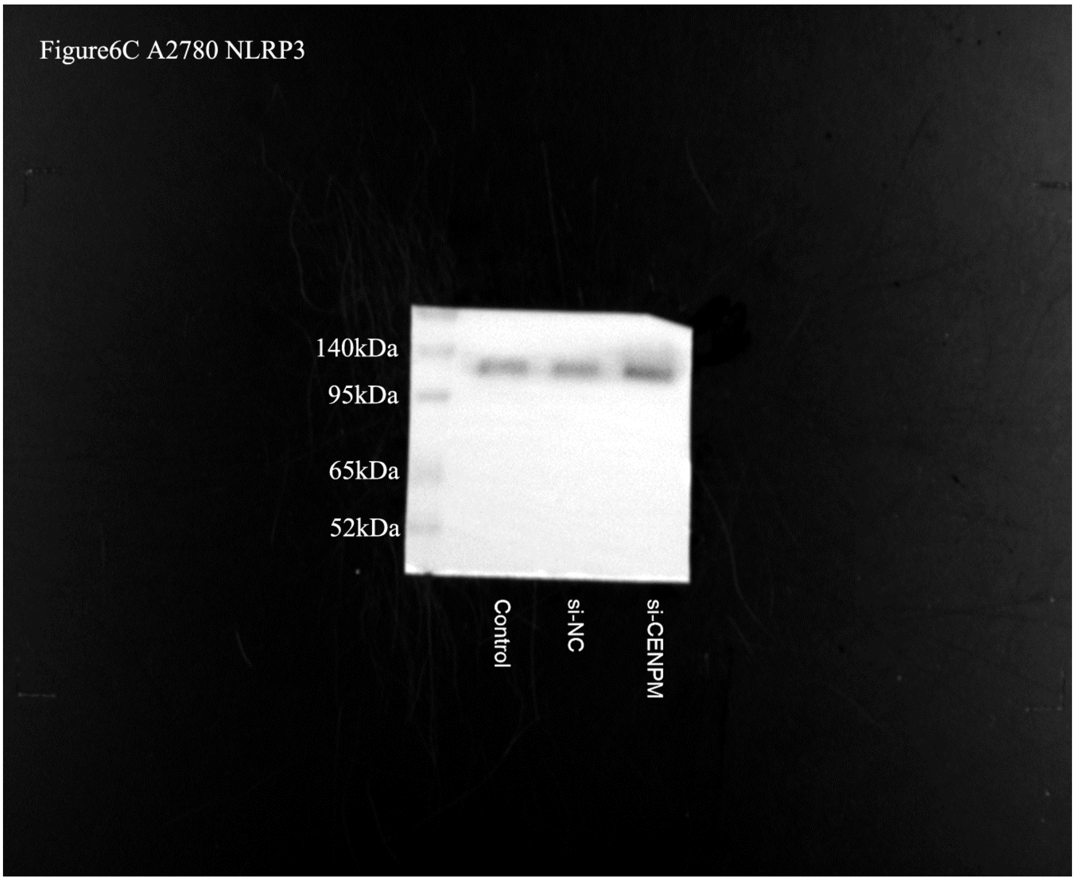

Figure 6C The β-actin protein band of A2780 cell

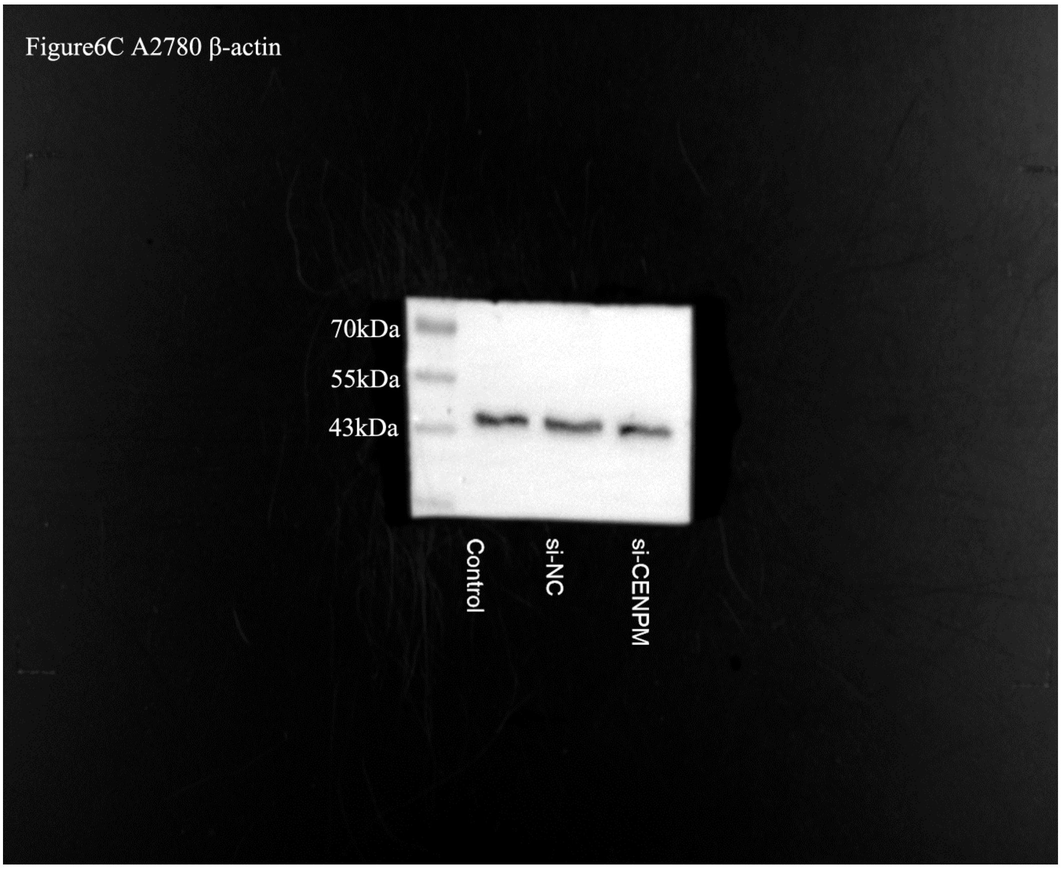

Figure 7F The E-cadherin protein band

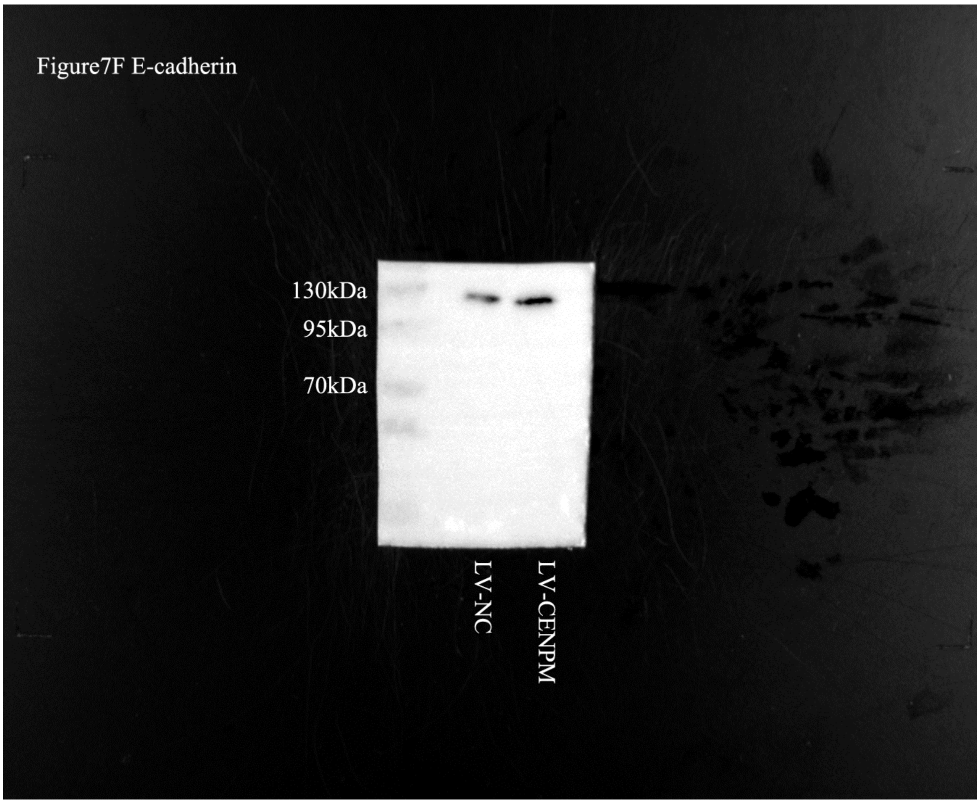

Figure 7F The N-cadherin protein band

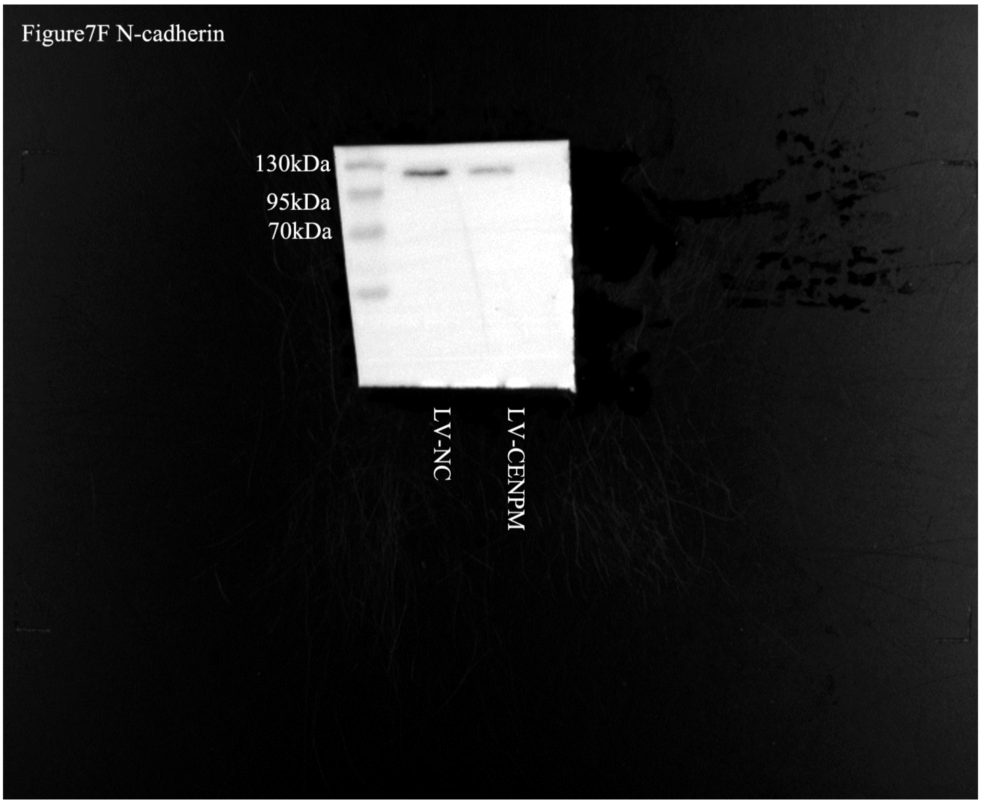

Figure 7F The vimentin protein band

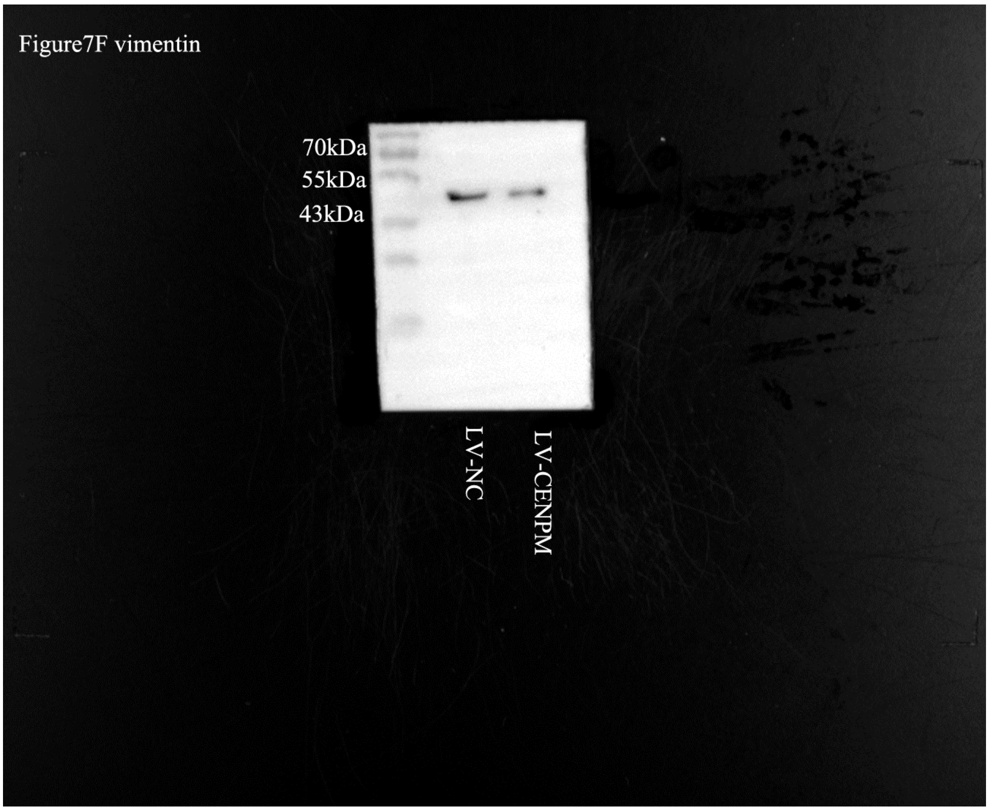

Figure 7F The β-actin protein band

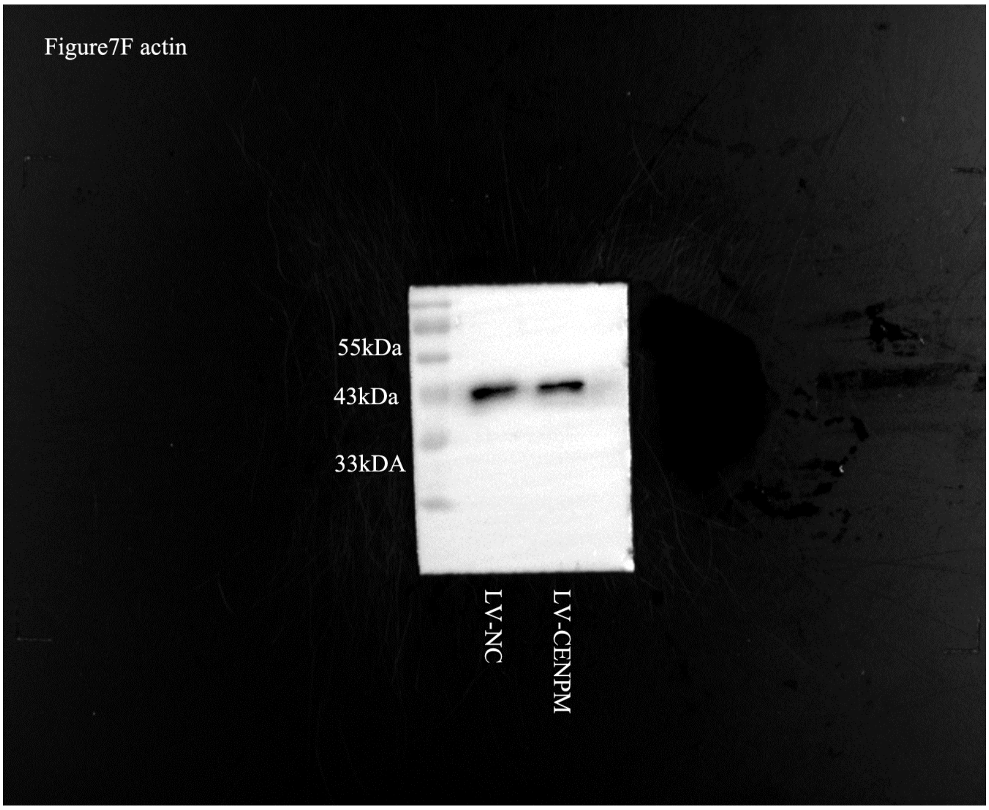

Figure 7G The CENPM protein band

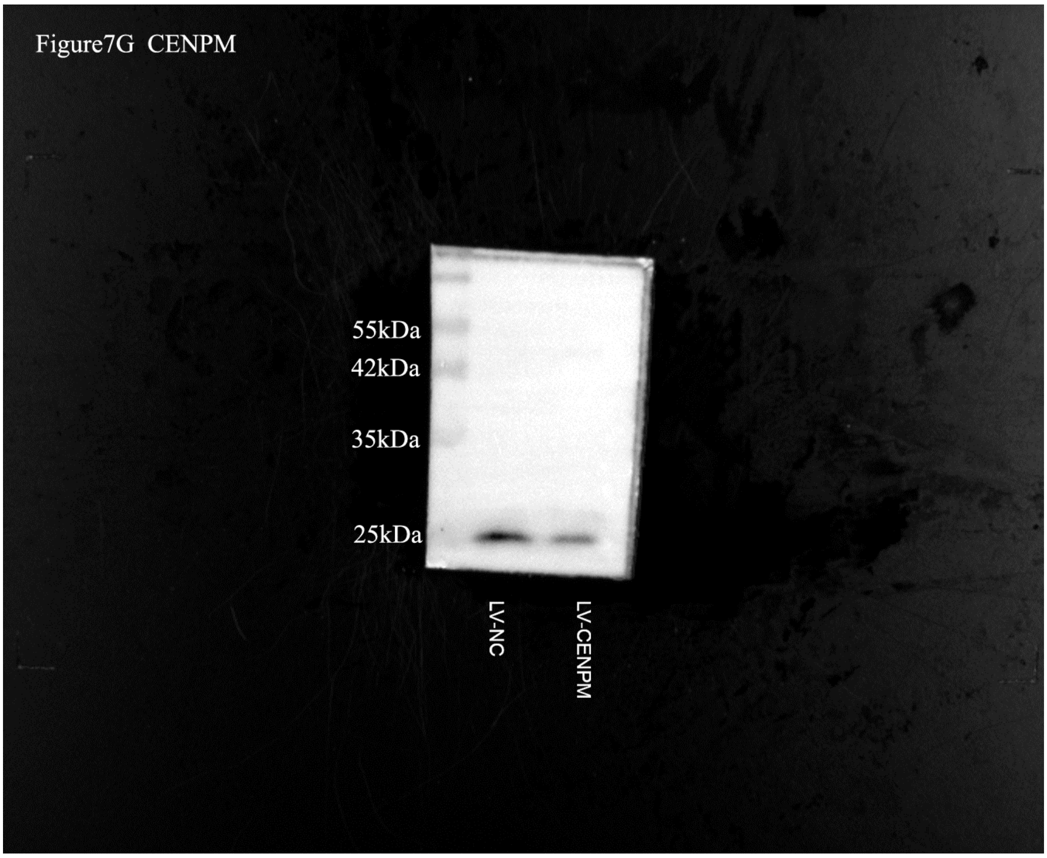

Figure 7G The Caspase-1 protein band

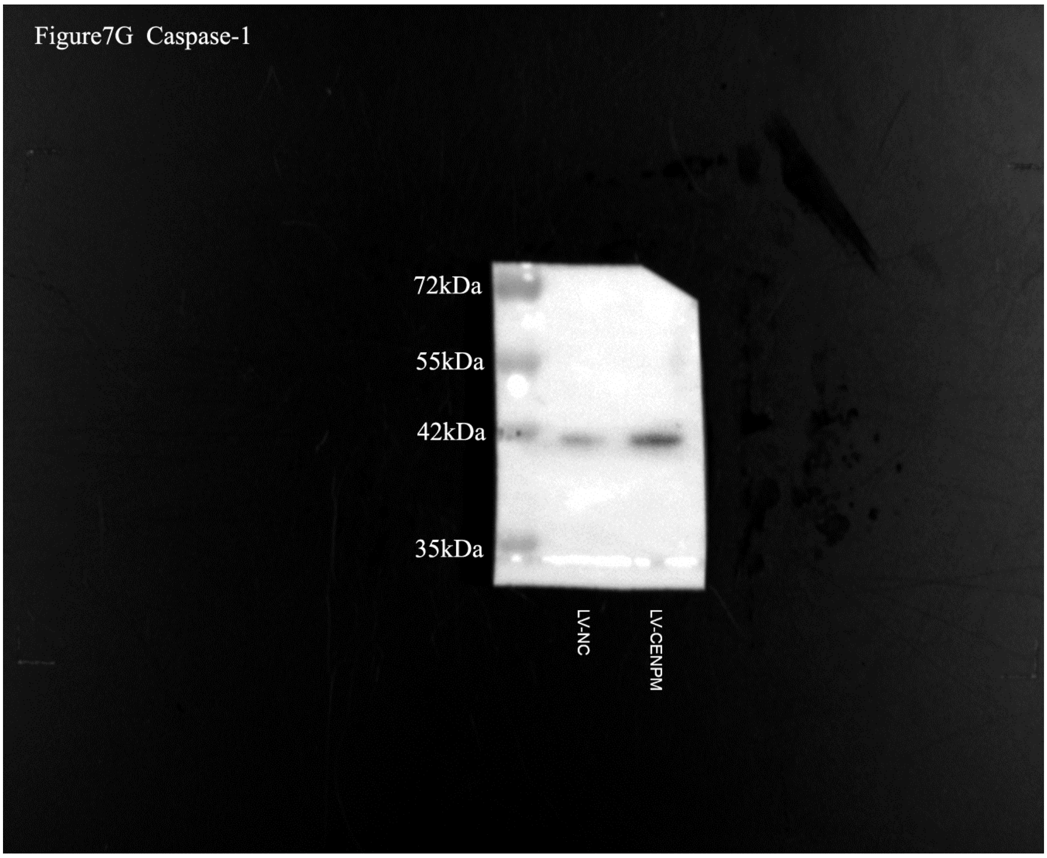

Figure 7G The GSDMD protein band

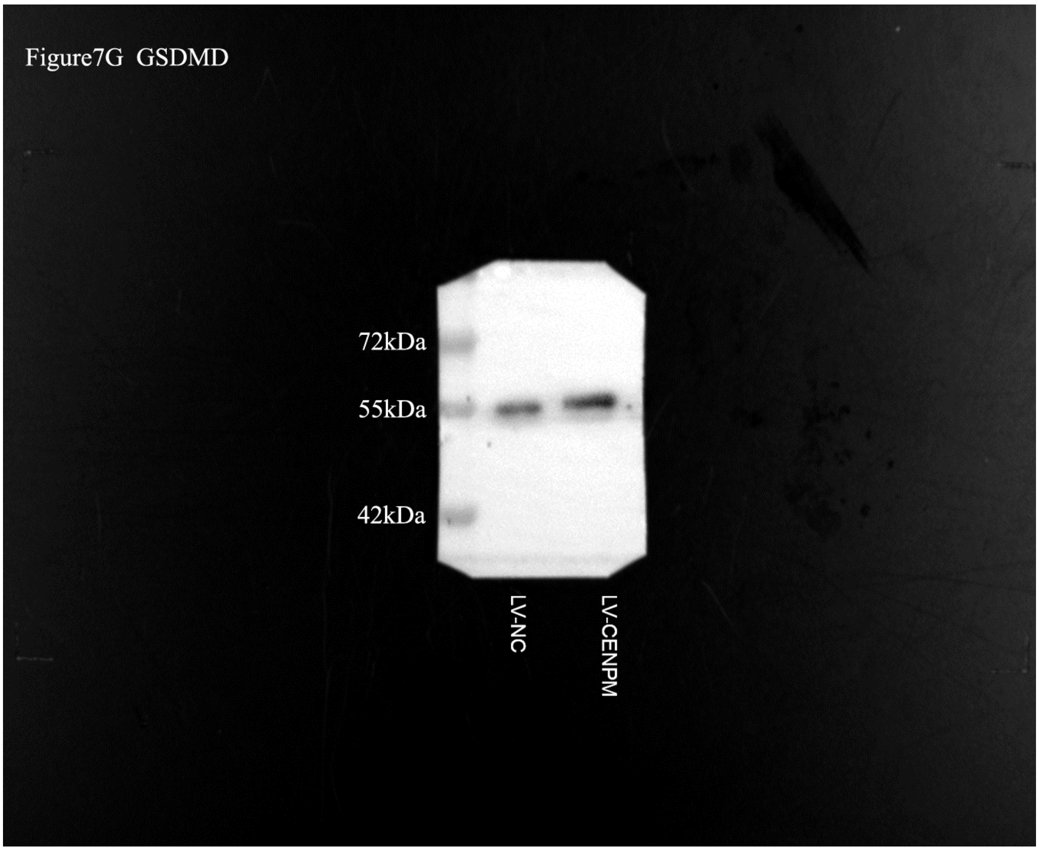

Figure 7G The NLRP3 protein band

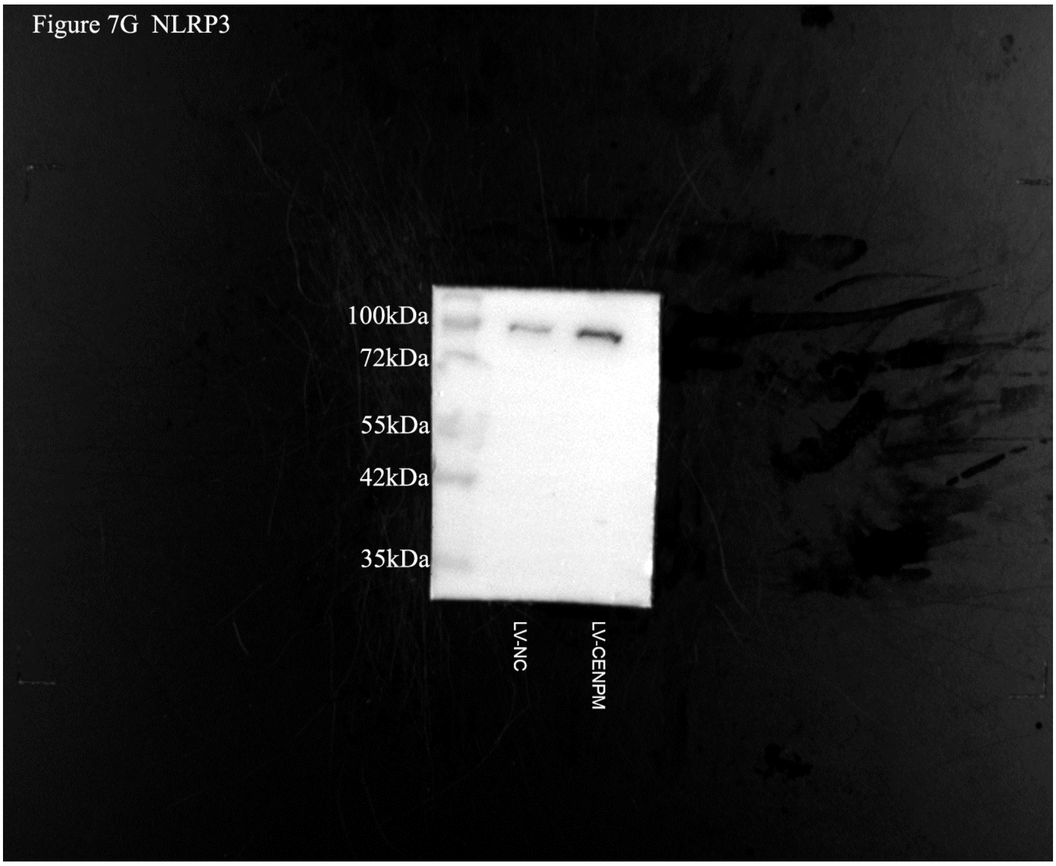

Figure 7G The β-actin protein band

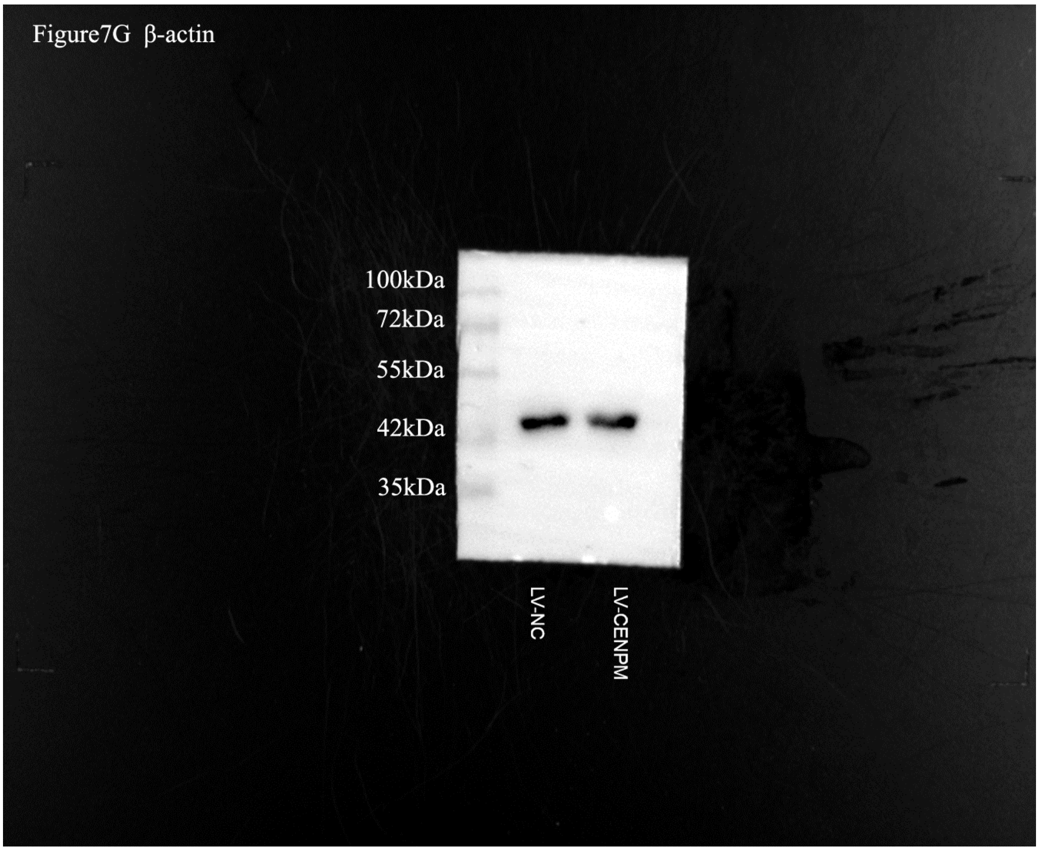

Figure 8A The cGSA protein band

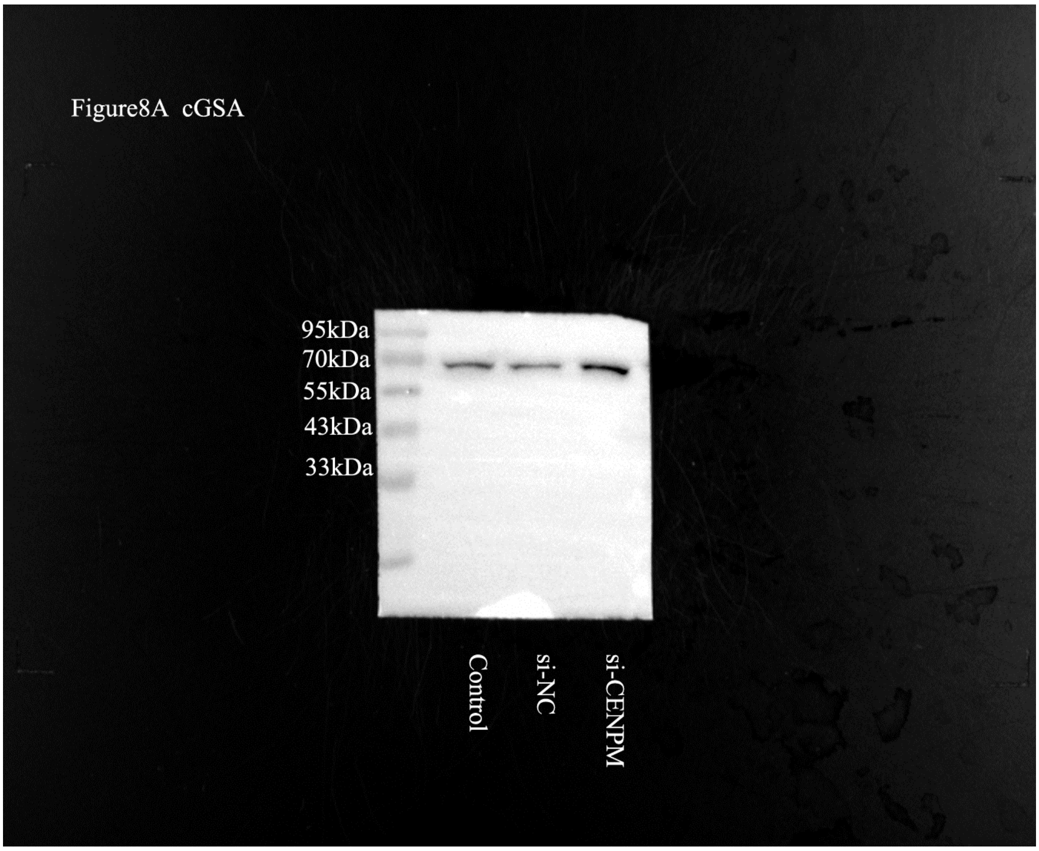

Figure 8A The p-STING protein band

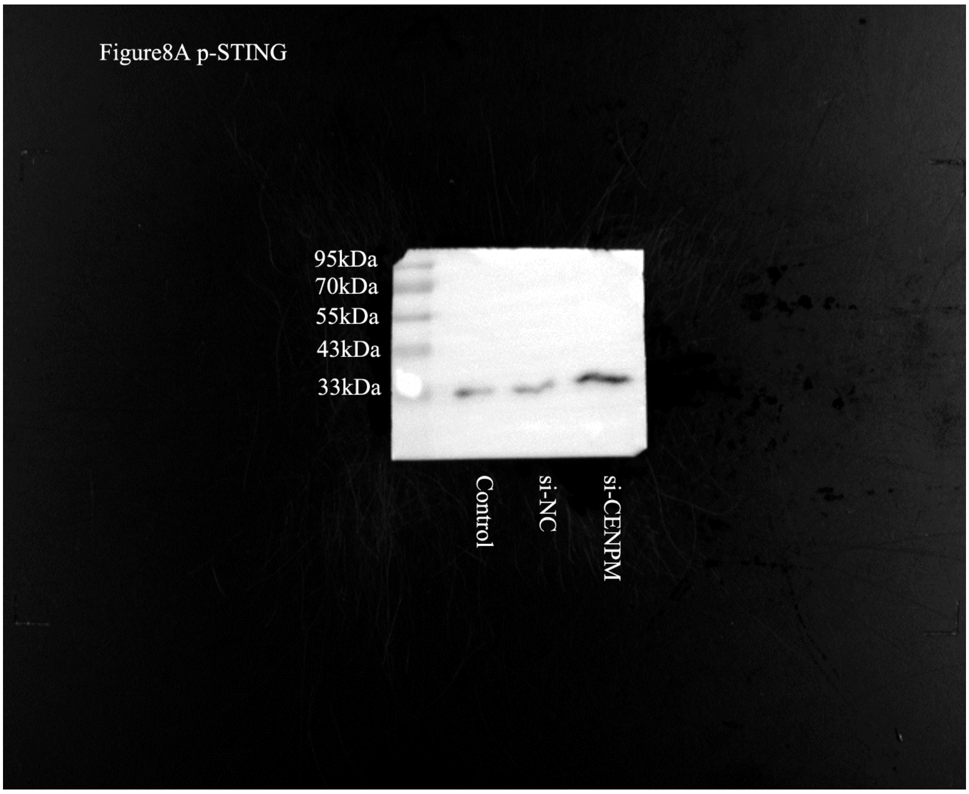

Figure 8A The STING protein band

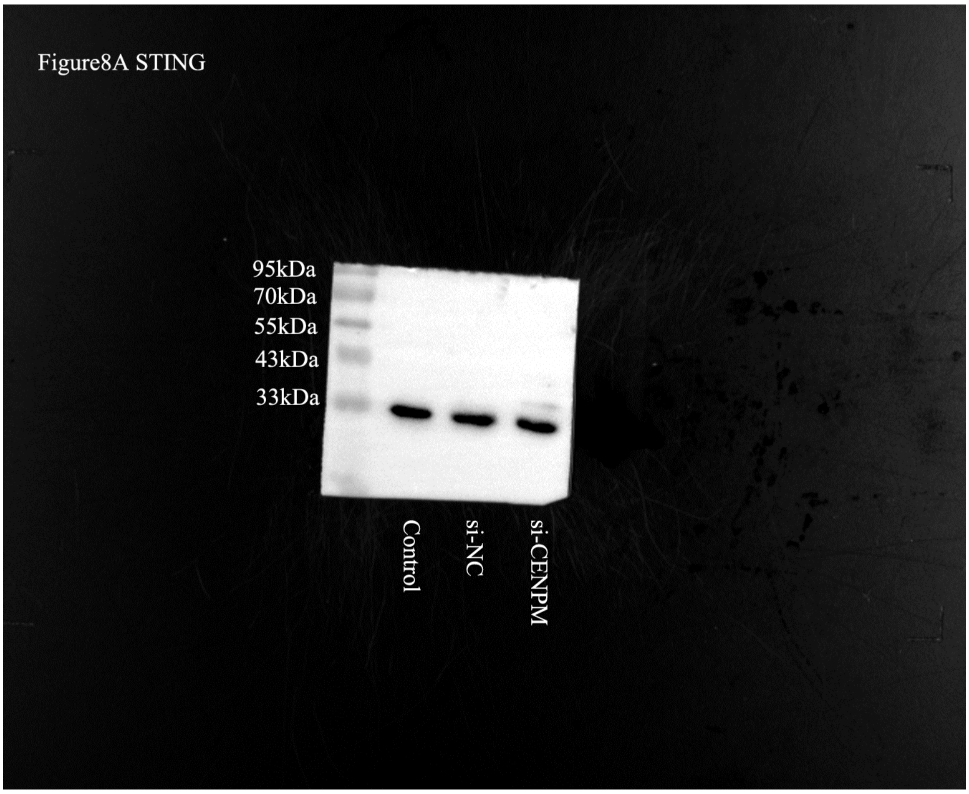

Figure 8A The β-actin protein band

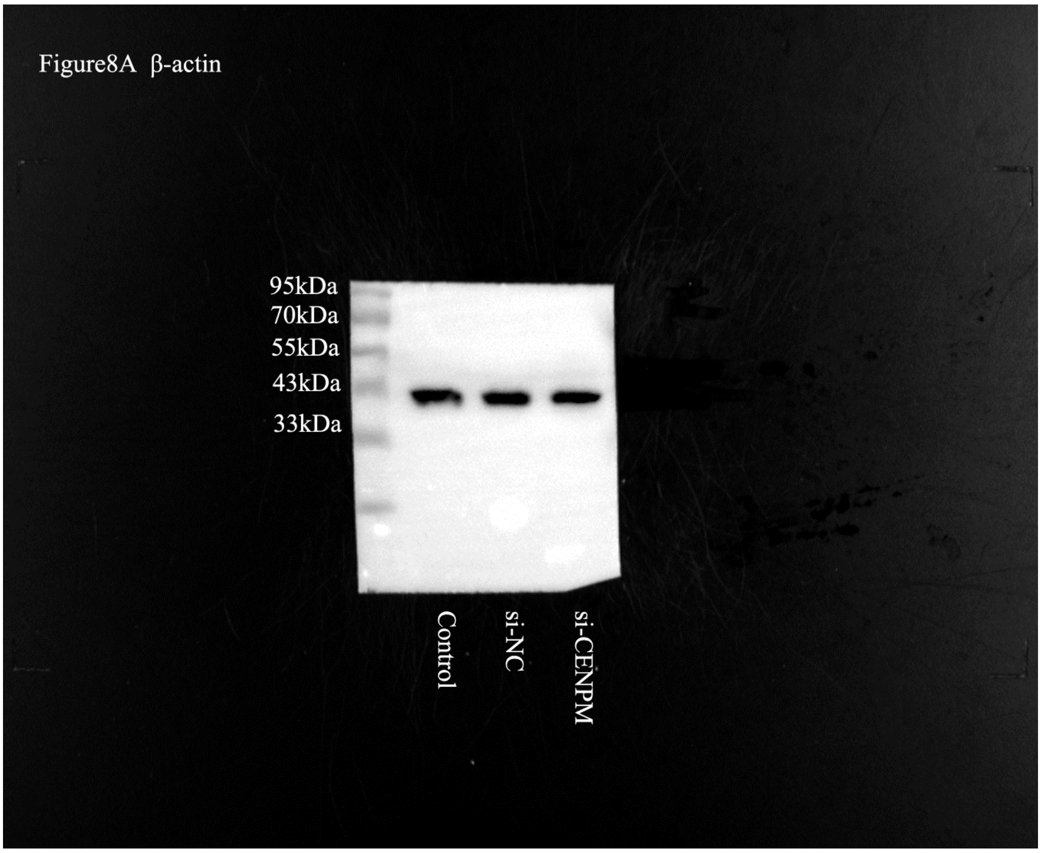

Supplement: Supplementary file 2 — Supplementary Material 2. [file 12885_2024_12296_MOESM2_ESM.pdf]
